# Species sorting shapes the divergence of a traditional fermented dairy-derived bacterial community with repeatable functionality during propagation with alternative substrates

**DOI:** 10.1007/s11274-026-04830-3

**Published:** 2026-04-28

**Authors:** Shepherd Nehanda, Anna Y. Alekseeva, Oscar van Mastrigt, Justin Chileshe, Bas J. Zwaan, Eddy J. Smid, Sijmen E. Schoustra

**Affiliations:** 1 Department of Biomedical Sciences, National Health Research and Training Institute (Formerly Tropical Diseases Research Centre), Ndola, Zambia; 2 Laboratory of Genetics and Laboratory of Food Microbiology, Wageningen University and Research, Wageningen, The Netherlands; 3 Department of Food Science and Nutrition, The University of Zambia, Lusaka, Zambia

**Keywords:** Adaptation, Diversity, Infant formula, Lactic acid bacteria, Natural microbial community, Selection

## Abstract

Species sorting underlies changes in microbial community composition under varying environments, yet predicting the species diversity and their functional outcomes when exposed to novel conditions remains challenging. We explored this using mabisi, a Zambian traditional fermented milk, by propagating a shared starting mabisi microbial community across five novel substrates - raw bovine milk (control), low-fat milk, full-cream milk, and the infant formulas F100 and S26 – under static conditions and fixed dilution for ~ 33 generations, at three rural farm sites. The microbial community composition was profiled through 16 S rDNA sequencing, and community-level functioning through volatile organic compounds, pH, and consistency. We observed a substrate-driven divergence of microbial communities, with low-fat milk enriched with *Acetobacter*, full-cream milk enriched with *Lactococcus*, and S26 enriched with *Lactiplantibacillus* and *Leuconostoc*. This pattern exhibited a temporal community succession but remained consistent between sites. Similarly, the community-level functionality diverged but remained largely repeatable to properties known for mabisi. Our findings suggest that novel environments drive a species sorting process in natural microbial communities, and this process persists over time. Additionally, the repeatability of its community-level functionality points to underlying functional redundancy within diverse and successive microbial communities. Our study not only advances the understanding of how microbial communities adapt to environmental changes but also provides a basis for harnessing the mabisi-derived microbial community for broad biotechnological applications, particularly supporting the feasibility and effectiveness of its use in an in-house formulated F100 infant formula for treating malnourished children in developing regions.

**Supplementary Information:**

The online version contains supplementary material available at 10.1007/s11274-026-04830-3.

## Introduction

Under the conceptual frameworks of microbial community assembly (Vellend 2010), species sorting is generally assumed to drive microbial community composition based on individual species’ differential sensitivity traits to environmental selection (Blasche et al. 2021; Li and Ma 2020; Nemergut et al. 2013), as opposed to a neutral process that assumes ecological equivalence (Sloan et al. 2006; Zhou et al. 2013). Species sorting is synonymous with the deterministic process, niche partitioning, and environmental filtering. Its role in governing the assemblage of microbial communities in natural habitats is critical for ecosystem functioning, ranging from organic matter decomposition in the soil (Maron et al. 2018); promoting health in the human gut (Rautmann and De La Serre 2021; Selber-Hnatiw et al. 2017); to contributing beneficial properties in fermented foods (Smid and Lacroix 2013). Yet, microbial communities are frequently faced with environmental changes in their natural habitats, which may consequently affect the prediction of their composition and functioning over time (Philippot et al. 2021). Under this topic, one of the questions is how species sorting sustains natural microbial communities and their functionality under novel environments in time and space.

Studies investigating associations between environmental variation and microbial community composition have gained traction. Recently, it was revealed that spatial variability plays a significant role in shaping microbial community diversity, primarily attributed to location-specific environmental factors, including micronutrient availability (Liao et al. 2016; Mandakovic et al. 2018; Zhang et al. 2020). Similar patterns have been observed in dairy ecosystems, where microbial community composition varies by location (Kochetkova et al. 2022; Moonga et al. 2020) and is linked to the location-specific environmental dynamics (Gobbetti et al. 2018; Quintana et al. 2020). Many of these studies, however, do not explicitly test the principle of the species sorting process, as they lack systematic tracking of a defined initial community (Johansen et al. 2019; Langenheder and Székely 2011). Moreover, natural systems are generally highly dynamic, and it is difficult to separate short-term fluctuations (e.g., diurnal temperature changes) from long-term shifts, and later on discern the sequence and relative influence of these environmental changes across sites over time (Hillebrand and Kunze 2020; Rodriguez-Ramos et al. 2021). These can potentially act as confounders, making it challenging to disentangle the specific mechanistic process of species sorting and to predict its role in sustaining microbial community diversity in natural habitats.

Furthermore, reconciling the species sorting process and microbial community functioning has remained inconclusive. For instance, species asynchrony was shown to be responsible for stabilizing community functions through the recruitment of alternative taxa to perform functions at different time scales (Wagg et al. 2021). Some studies, however, argue that changes in microbial diversity affect specific system functions (Chen et al. 2021; Peter et al. 2011). Additionally, other studies that have explored community assembly have either overlooked the linkage to functional outcomes (Dong et al. 2021) or restricted to the screening of genetic potential through transcriptomic (rRNA) analysis in artificially assembled communities (Blazewicz et al. 2013), which may not always be translated to functional metabolites in situ, i.e., in natural settings. With the advent of meta-omics (Ferrocino et al. 2023), it is now plausible to mechanistically complement species sorting processes with functional insights by associating the microbiome with metabolomic data. However, this approach remains challenging with complex field study models. Therefore, simpler and suitable study models are warranted to effectively test these processes and derive potential generalizable inferences.

In this study, we leverage mabisi, a traditional fermented milk beverage found in Zambia (Schoustra et al. 2013), to address how species sorting mechanisms govern a natural microbial community assembly and maintain their functionality upon exposure to novel environments in time and space. Mabisi microbial communities are self-assembled during the traditional fermentation, hence, they possess features representative of a natural community model system (Alekseeva et al. 2021; Blasche et al. 2017). These communities are dominated by beneficial bacterial guilds, including *Lactococcus*, *Acetobacter*, *Leuconostoc*, *Lactiplantibacillus*, *Paucilactobacillus*, and *Lacticaseibacillus* (Leale et al. 2023; Moonga et al. 2020), which underlie measurable functional parameters such as changes in pH, volatile organic compounds (VOC), and consistency during the culture processes (Groenenboom et al. 2022; Moonga et al. 2021). Thus, mabisi attracts usage as a model to study microbial community assembly, such as when communities from different origins were transferred into a shared but novel environment, and revealed patterns of coalescence and divergence (Groenenboom et al. 2022).

**Fig. 1:**
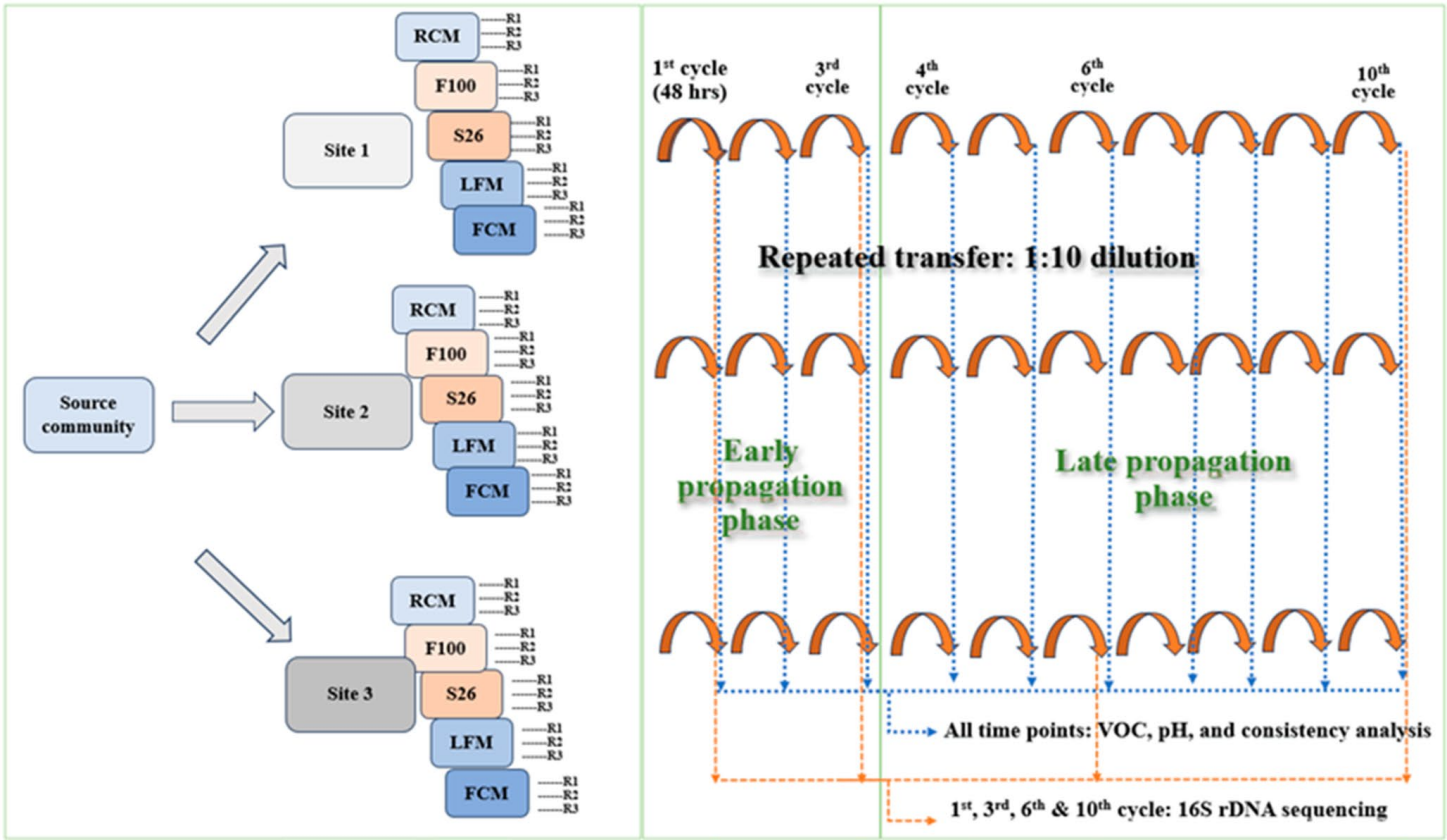
The field experimental setup. Mabisi microbial communities from a common source (starting community) were inoculated (1:10 dilution) into five milk substrates: raw cow milk (RCM), F100 infant formula (F100), S26 infant formula (S26), ultra-high temperature low-fat milk (LFM) and ultra-high temperature full cream milk (FCM), with 3 replicates per substrate at each of three farm sites (site 1, site 2 and site 3). Propagation was conducted by serially transferring 1:10 of cultures into fresh substrates every 48 h for 10 cycles

Under natural conditions at three rural farm sites in Zambia, we exposed a shared starting mabisi microbial community to five bovine-derived milk substrates. Among these, raw cow milk served as a control, given that it is the usual substrate for mabisi processing (Schoustra et al. 2013). Moreover, ultra-high temperature low-fat milk, ultra-high temperature full-cream milk, and the infant formulas F100 and S26 represented novel environments that varied in pH, pretreatment methods, nutritional properties, and purposes (Kunda et al. 2015; Nyirenda et al. 2009; Park et al. 2012). Each substrate was inoculated with a shared starting mabisi microbial community at a fixed dilution with three replicates, and repeatedly propagated over 10 cycles, categorized arbitrarily as early (1–3 cycles) and late (4–10 cycles) propagation phases (Fig. 1). Specifically, the bacterial community changes were analyzed by 16 S rDNA sequencing and diversity (alpha and beta) metrics, while the community-level functionality was measured through monitoring volatile organic compounds, pH and consistency. This experimental setup enabled us to optimize the occurrence of the selection process in situ, that is, under natural field conditions. With this, we could simultaneously track how species sorting shaped the microbial community diversity and their functionality in time and space. We hypothesize that propagation within novel environments leads to a detectable species sorting process, revealing a substrate-driven divergence of bacterial community composition and functionality over time across sites. Alternatively, if species sorting is weaker, the resultant microbial community diversity and functional patterns would exhibit random radiation with no discernible differences between substrates. Our study informs fundamental ecological insights on the microbial community responses to environmental changes, and also provides a basis for predicting how a natural mabisi microbial community can be diversified for biotechnological applications to enhance novel fermented food ingredients.

## Methods

### Propagation experiment in the field

Between November to December 2020, a field experiment was set up at three nearby farms in the Copperbelt Province, Zambia (Latitude − 12.9833^0^S, longitude 28.6333^0^E). The sites were located within a radius of approximately two Km for logistical convenience. Each site had unique in-house mabisi processing conditions; site 1 involved incubation in an outdoor and elevated space; site 2 involved incubation on a wooden platform in an enclosure; and site 3 involved incubation on the floor, but also in an enclosure. The study utilized five substrates: raw cow milk (RCM) serving as a control (Schoustra et al. 2013), ultra-high temperature low-fat milk (LFM), ultra-high temperature full-cream milk (FCM), F100 infant formula (F100), and S26 infant formula (S26), representing novel environments differing in pretreatment and nutritional parameters (Table 1) (Kunda et al. 2015; Nyirenda et al. 2009; Park et al. 2012). The RCM was sourced and homogenized from three local dairy farmers. The F100 was prepared according to a World Health Organization (WHO) guided in-house protocol (World Health Organisation, 1999), while S26, LFM, and FCM were sourced ready for use from a local supermarket. The milk substrates were inoculated with a pooled mabisi sample, representing a source community, in the ratio of 1:10, in a total volume of 1 L. There were three replicates for each milk substrate at each farm site. The preparation was left to ferment for a complete cycle of 48 h. Analogous to an evolution experiment, a portion of the fermented products was serially transferred into a fresh set of its respective substrates in the same ratio over time: designated arbitrarily as early propagation phase (1st – 3rd cycle) and late propagation phase (4th – 10th cycle) for a total of 10 cycles (~ 33 bacterial generations). Culture products were sampled in 50 mL clean Falcon tubes at the end of each cycle, placed in a cooler box with ice, and transported to the central laboratory at the National Health Research and Training Institute (NHRTI) (formerly the Tropical Diseases Research Centre) for further analysis and storage at −20 °C. A summary of the experimental setup is depicted above (Fig. 1).

**Table 1:** Summary of nutritional parameters for substrates utilized in this study

Substrate	Nutritional parameter (per 100 g edible portion)													Reference
	Energy (Kcal)	Protein (g)	Fat (g)	Carbohydrate (g)	Ca^2+^(mg)	Fe (mg)	Zn (mg)	Vitamins						
								A (µg)	B1 (mg)	B2 (µg)	B3 (mg)	C (mg)		
F100	100	6	12.2	4.2	140.9	1.0	5.0	-	0.2	0.7	2.4	23.4	(Park et al., 2012)	
S26	570	12.5	30	-	370	10.6	-	661	-	0.92	8.3	48	(Nyirenda et al., 2009)	
RCM	64	3.3	3.6	4.7	120	0.1	-	45	0.04	0.15	0.1	-	(Nyirenda et al., 2009)	
FCM	62.4	-	3.2	-	119	-	-	-	-	-	-	-	Package labels*	
LFM	37.44	-	0.5	-	123	-	-	-	-	-	-	-	Package labels*	

‘-’ means value missing and ‘*’ details obtained from manufacturer’s labels on the container. Substrates are represented by F100 infant formula (F100), S26 infant formula (S26), raw cow milk (RCM), ultra-high temperature low-fat milk (LFM), and ultra-high temperature full-cream milk (FCM)

## Composition and diversity of microbial communities

### DNA extraction

For bacterial community profiling, mabisi samples from the 1 st, 3rd, 6^th,^ and 10th cycles of fermentation were selected. The DNA extraction was performed according to the previously described protocol (Schoustra et al. 2013), with minor modifications. Briefly, 1 mL mabisi sample was centrifuged in a 2 mL screw capped tube at 12,000 rpm for 2 min. The pellets were resuspended in a cell digestion solution containing 64 µl of a 0.5 M EDTA, 160 µl nuclei lysis reagent (Promega), 5 µl RNase (10 mg/mL), 120 µl of lysozyme (10 mg/mL), and 40 µl pronase E (20 mg/mL) reagents. The mixture was incubated automated heating block for 60 min at 37 °C while shaking at 350 rpm. The tubes were subjected to bead beating with 2 scoops of sand-sized beads (Sigma, Germany) following an in-house *Lactoccocus* lysis protocol, while cooling on ice in between. After, 400 µl of ice-cold 0.5 M ammonium acetate (Sigma Aldrich) was added, mixed, and incubated for 15 min at room temperature. The tubes were centrifuged at 13,000 rpm for 4 min. Later, 700 µl of the supernatant was transferred into a sterile 1.5 mL capped cryovial, and an equal volume of molecular grade phenol pH 8.0 (Sigma Aldrich) was added. The tubes were spun at 12,000 rpm for 6 min, and 350 µl of the supernatant was transferred into a new 1.5 mL cryovial tube. An equal volume of chloroform (Sigma Aldrich) was added and centrifuged at 12,000 rpm for 2 min to remove the phenol. Thereafter, 300 µl of the supernatant was transferred into a new 1.5 mL cryovial tube to which 400 µl of isopropyl alcohol (Sigma Aldrich) was added. The tubes were placed in a −20 °C freezer overnight to precipitate the DNA. After, the tubes were centrifuged for 15 min at 13,000 rpm at 4 °C and the DNA pellets were washed with 1 ml ice-cold 70% ethanol by carefully inverting the tubes 10 times, followed by centrifugation at 12,000 rpm at 4 °C for 10 min. The wash was repeated. The supernatant was carefully decanted, and the tubes were left to dry for 5 min the heating block at 37 °C. After, the DNA was eluted by adding 20 µl of low EDTA elution buffer (10 mM Tris, bring to pH 8.0 with HCL; 1 mM EDTA) (Sigma Aldrich). A NanoDrop™ ND-2000 and Qubit ^TM^ 4 fluorometer (Thermal Fisher Scientific, UK) was used to check for the DNA quantity and quality and stored at −20 °C until further needed.

### 16 S rDNA sequencing

To profile the bacterial community structure and their temporal dynamics following the experimental treatments described earlier, the extracted DNA samples were sent for 16 S rDNA sequencing to Novogene, United Kingdom. Polymerase chain reaction (PCR) was used for library preparation using 341 F CCTAYGGGRBGCASCAG and 806R GGACTACNNGGGTATCTAAT universal primers targeting the 16 S rDNA V3-V4 region, according to the previously described protocol (Schoustra et al. 2013), with modifications. Briefly, the PCR products were purified, end-repaired, A-tailed and ligated with Illumina adapters, and sequenced on the NovaSeq PE250 platform (Lianchuan Biotechnology Company Limited, Hangzhou, China) to generate 250 bp paired end raw reads. The barcode and primer sequences were truncated and FLASH (v 1.2.11) applied to merge the reads. The reads underwent filtering, denoising, removal of chimera, and generation of amplicon sequence variants (ASV) with DADA2 (V 1.14.1), with parameters set as follows: trimLeft = c(17,21), truncLen = c(240, 160), maxN = 0, maxEE (2.2), and truncQ = 2 (Callahan et al. 2016). The ASVs were annotated using a publicly available SILVA database (V 138.2): silva_nr99_v138.2_toGenus_trainset.fa.gz (Wambua 2025). Then, the ASVs were normalized by rarefying with random repeated sampling of samples to a minimum sequence reads of 19,534 without replacement, nonbacterial taxa excluded, and a cut-off relative abundance of 0.25% applied in at least each sample to exclude singleton or spurious taxa before downstream analysis (Reitmeier et al. 2021), with R (version 4.5.0) and phyloseq package (version 1.52.0).

## Microbial community-level functionality

### Volatile organic compounds

Volatile organic compounds (VOC) were also used as a proxy measure for the metabolic activity following propagation of mabisi microbial communities in varied milk substrates. The VOC were analyzed by Headspace-Solid Phase Microextraction Gas Chromatography-Mass Spectrometry (HS-SPME, GC-MS), Trace 1300 Gas Chromatograph (Thermo Fisher), TriPlus RSH autosampler (Thermo Fisher) and an ISQ QD mass spectrometer (Thermo Fisher) using an inhouse protocol, as previously described (Moonga et al. 2021). Briefly, 1mL of mabisi sample was injected into GC-MS labeled vials with caps fastened, and placed on the GC-MS platform to incubate at 60 °C for 20 min. The VOCs were allowed to vaporize and adsorb on the SPME fiber (Car/DVB/PDMS/Supelco) at 60 °C for 20 min. Next, the extracted VOCs were desorbed for 2 min onto a Stabilwax ^®^-DA column (30 m length, 0.25 mm ID, 0.5 μm df, Restek), PTV split-less mode at 250 °C for 5 min with helium gas as carrier at 1.5 ml/min. The GC oven was set at 40 °C for 2 min, raised to 240 °C with a slope of 10 °C/min, and kept at 240 °C for 5 min. Mass spectra data were collected over a range of m/z 33–250 in full scan mode with 3.0030 scans/sec. The obtained data were analyzed by Chromeleon ^®^ 7.2 software (Thermo Fisher) using the ICIS algorithm and the NIST main library for signal peak integration and compound hit annotation. The RSI match factor of > 750 was applied. The results were exported to Excel files for statistical analysis. Nine samples, spanning substrate type, propagation phases, and sites, showed no detectable values for all the tested VOCs and were therefore considered technical failures and excluded from downstream analysis.

### pH

The fermentation process was monitored by recording changes in pH at the initial time point, after 24 h and 48 h, respectively, using a digital pH meter (Jenway, UK). The pH probe was disinfected with 70% ethanol and rinsed in sterile water before and in-between measurements of each sample.

### Consistency

At the end of each fermentation cycle, consistency was measured by recording changes in viscosity of the samples using an Adam’s consistometer, according to the previously described protocol (Moonga et al. 2021). Briefly, fermented samples were decanted into a 17 mL capacity Adam’s consistometer cylinder placed on a graduated platform. The cylinder was lifted to allow the test material to spread freely for 30–60 s. The degree of spread was recorded in centimeters following pre-marked readings on the platform.

## Statistical analysis

The R statistical software (version 4.5.0) was used for all data analysis. The microbiome data were visualized by box plots, histographs, and non-metric multidimensional scaling plots. Furthermore, alpha (Chao1 and Shannon) and beta diversity metrics were applied with Wilcoxon rank-sum and analysis of group similarities (ANOSIM) tests, corrected by the Benjamin-Hochberg method, to determine how microbial community diversity varied by study parameters. Variability in dispersion of community composition within samples was analyzed using the Vegan package (version 2.7.2). Species differential analysis was further explored through a linear discriminant effective size (LEfSe) analysis using the microbiomeMarker package (version 1.13.2).

The VOC data were plotted with heatmaps and principal component analysis (PCA) for visualization and analyzed by permutation multivariate analysis of variance (PERMANOVA) with default free permutation of 999 after normalization using a log transformation and median scaling by compound (column). Variability in VOC dispersion within samples was also analyzed using the Vegan package (version 2.7.2). The pH and consistency were summarized by median, lower and upper quartiles, and visualized by violin plots. Kruskal-Wallis and Dunn tests, with the Benjamin-Hochberg adjusted method, were applied whenever appropriate to analyze the statistical differences between experimental parameters. The significance test was set at 0.05 for all analyses.

## Results

### Composition and diversity of microbial communities

**Fig. 2:**
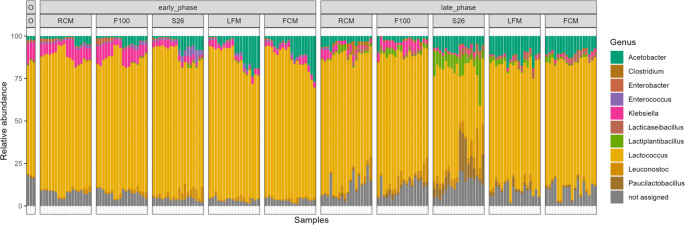
Distribution of propagated mabisi microbial community in different milk substrates over time. The y-axis represents the relative abundance (%) of each taxon in each sample, while the x-axis shows sample identities (Sample ID), faceted by propagation phases: starting mabisi microbial community (O), early phase (early_phase), and late phase (late_phase). Substrates are represented by raw cow milk (RCM), F100 infant formula (F100), S26 infant formula (S26), ultra-high temperature low-fat milk (LFM), and ultra-high temperature full-cream milk (FCM). The legend lists the most abundant genera; *Acetobacter* (dark teal), *Clostridium* (brownish orange), *Enterobacter* (warm terracotta), *Enterococcus* (soft lavender purple), *Klebsiella* (magenta), *Lacticaseibacillus* (mutated brick red), *Lactiplantibacillus* (bright olive green), *Lactococcus* (golden yellow), *Leuconostoc* (dark mustard yellow), *Paucilactobacillus* (earthly brown), and unassigned - referring to ASVs that could not be annotated in the silva database (medium gray)

A total of 4322 bacterial ASVs were obtained. At the phylum level of classification, these taxa were predominantly represented by the Bacillota, and this pattern was consistent with the starting mabisi microbial community (Fig S1). However, at the genus level, and specifically focusing on key lactic acid bacteria (LAB) and acetic acid (AAB) community members known for their important role in dairy ecosystems, the members, including *Lactococcus*, *Acetobacter*, *Leuconostoc*, *Lactiplantibacillus*, *Paucilactobacillus*,* and Lacticaseibacillus* were dominant in all substrate treatments at all farm sites over time (Fig. 2; Fig. S2).

### Alpha and beta diversity analysis of microbial communities

**Fig. 3:**
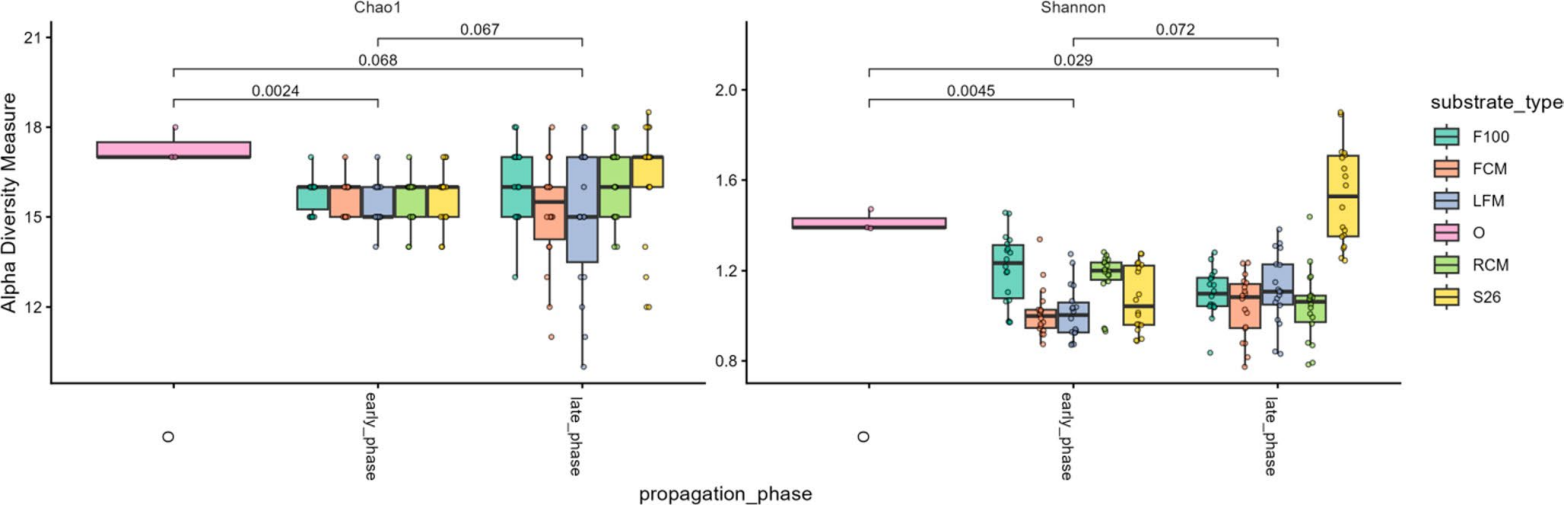
Alpha diversity of mabisi microbial community by substrate treatment over time. Chao1 (richness) and Shannon (incorporating both richness and evenness) diversity metrics were applied to assess microbial community alpha diversity following propagation of a shared starting mabisi microbial community in varied milk substrates. The y-axis represents the diversity indices (Chao1 and Shannon), while the x-axis represents propagation phases: starting mabisi microbial community (O), early phase (early_phase), and late phase (late_phase). Box plots display the median, interquartile range, whiskers, and outliers. Dots represent individual sample data from each substrate treatment at each propagation phase. The legend indicates the substrate treatment: F100: F100 infant formula (medium aquamarine), FCM: ultra-high temperature full-cream milk (coral), LFM: ultra-high temperature low-fat milk (dark pastel blue), O: starting mabisi microbial community (middle purple), RCM: raw cow milk (yellow green), and S26: S26 infant formula (golden). The pairwise analysis of the differences in Chao1 and Shannon diversity indices of substrates between the propagation phases was analyzed by the Wilcox test, with numbers in the comparisons representing statistical *p*-values

To gain further insights into whether propagation of a shared starting mabisi microbial communities in varied substrates exerted diversity changes in the microbial community composition at different farm sites over time, alpha and beta diversity analysis was applied. The alpha diversity was analyzed by Chao1 (richness) and Shannon (richness and evenness) indices, with raw cow milk (RCM) used as a reference (the usual substrate for mabisi microbial community). Relative to the RCM, all substrate treatments did not show significant differences in the resultant community richness (*p* > 0.05) (Fig. 3; Table S1) or across sites (*p* > 0.05) (Fig. S3; Table S1). Also, the richness did not differ between the early and late propagation phases (*p* > 0.05) (Fig. 3; Table S1). However, both the richness and evenness of the propagated microbial community from FCM (*p* = 0.018) and S26 (*p* = 0.004) based substrate treatments increased significantly, but not in the F100 and LFM-based treatments (*p* > 0.05) (Fig. 3; Table S1) relative to RCM. Additionally, the richness and evenness did not differ between the early and late propagation phases (*p* = 0.07) or farm sites (*p* > 0.05) (Fig. S3; Table S1). Overall, this suggests that substrate variation constituted niche division, shaping differences in the community alpha diversity, while propagation phases or site differences did not.

**Fig. 4:**
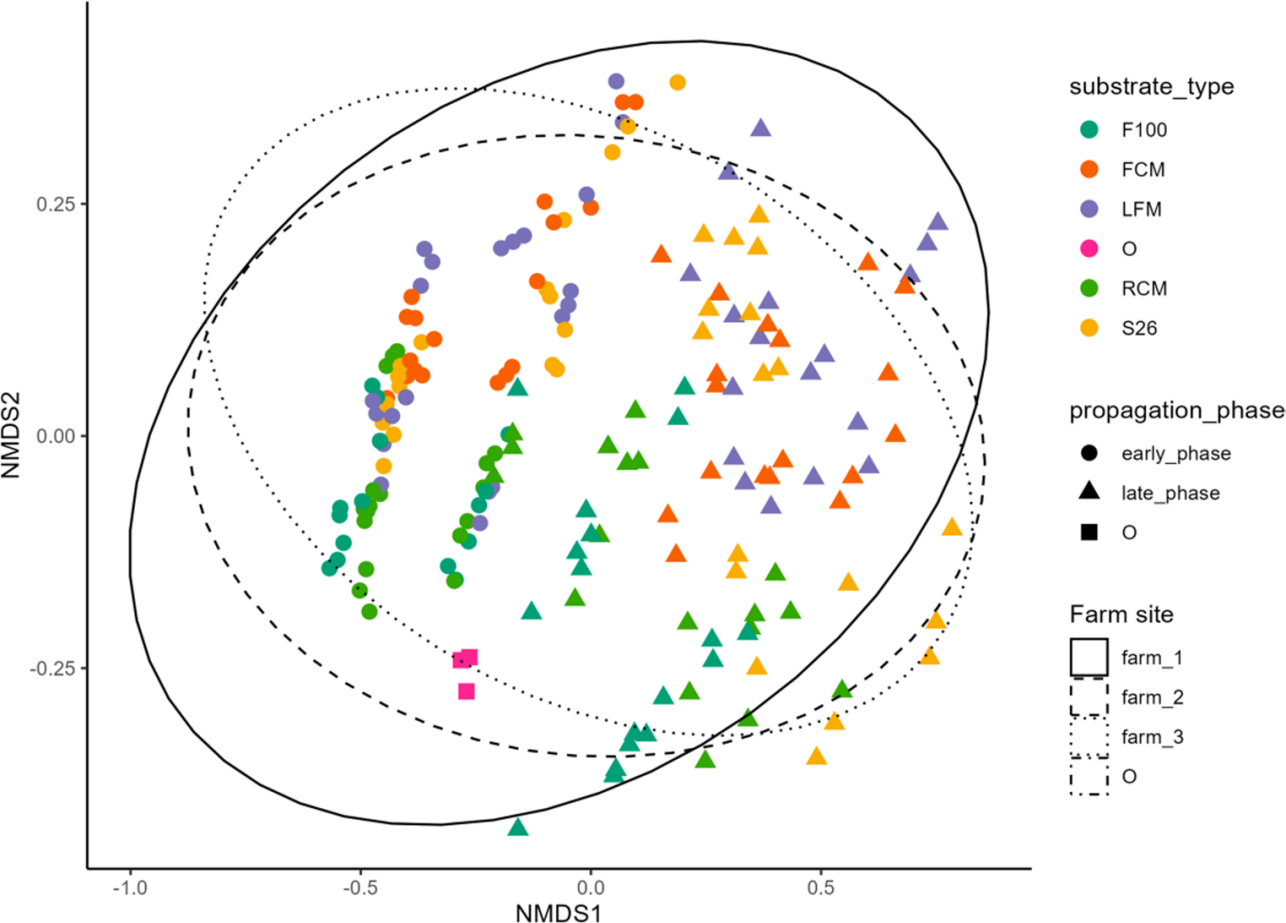
Dissimilarity in microbial communities by non-metric multidimensional scaling (NMDS). The NMDS was applied to show the shifts in microbial community diversity following the propagation of mabisi microbiota through varied milk substrates over time. Substrates are distinguished by colors - F100: F100 infant formula (teal), FCM: ultra-high temperature full-cream milk (orange), LFM: ultra-high temperature low-fat milk (purple), O: starting mabisi microbial community (magenta), RCM: raw cow milk (green), S26: S26 infant formula (yellow). Propagation phases are indicated by shape – early_phase: early phase (filled circle), late_phase: late phase (filled triangle point up), and O: starting mabisi microbial community (filled square). Ellipses represent farm sites at 95% confidence interval: farm 1 (solid), farm 2 (dashed), farm 3 (dotted), and O not shown due to low data points (dash-dotted)

To assess the dissimilarity in the community composition following the repeated propagation of mabisi microbial communities across substrates and sites over time, a non-metric multidimensional scaling (NMDS) with permutation of multivariate analysis (PERMANOVA) was conducted. The microbial community significantly differed in its composition mediated by the substrate variation (ANOSIM: *R* = 0.16, *p* = 0.001), propagation phases (ANOSIM: *R* = 0.40, *p* = 0.001), and site differences (ANOSIM: *R* = 0.03, *p* = 0.014) (Fig. 4, Table S2).

Furthermore, beta dispersion analysis was conducted to determine the homogeneity of variability in community composition within samples following substrate treatment between propagation phases. This analysis revealed that microbial community composition variability within the substrate group did not differ during the early phase (F = 1.96, *p* = 0.11) (Table S3). However, community variability was significantly different between substrates in the late phase of propagation (F = 9.44, *p* = 0.001) (Table S3). This demonstrated that microbial communities exhibited a substrate-specific divergence, particularly during the early phase of propagation, but this pattern became more heterogeneous during the late phase of propagation.

### Linear discriminant effect size (LEfSe) analysis of differential marker species

**Fig. 5:**
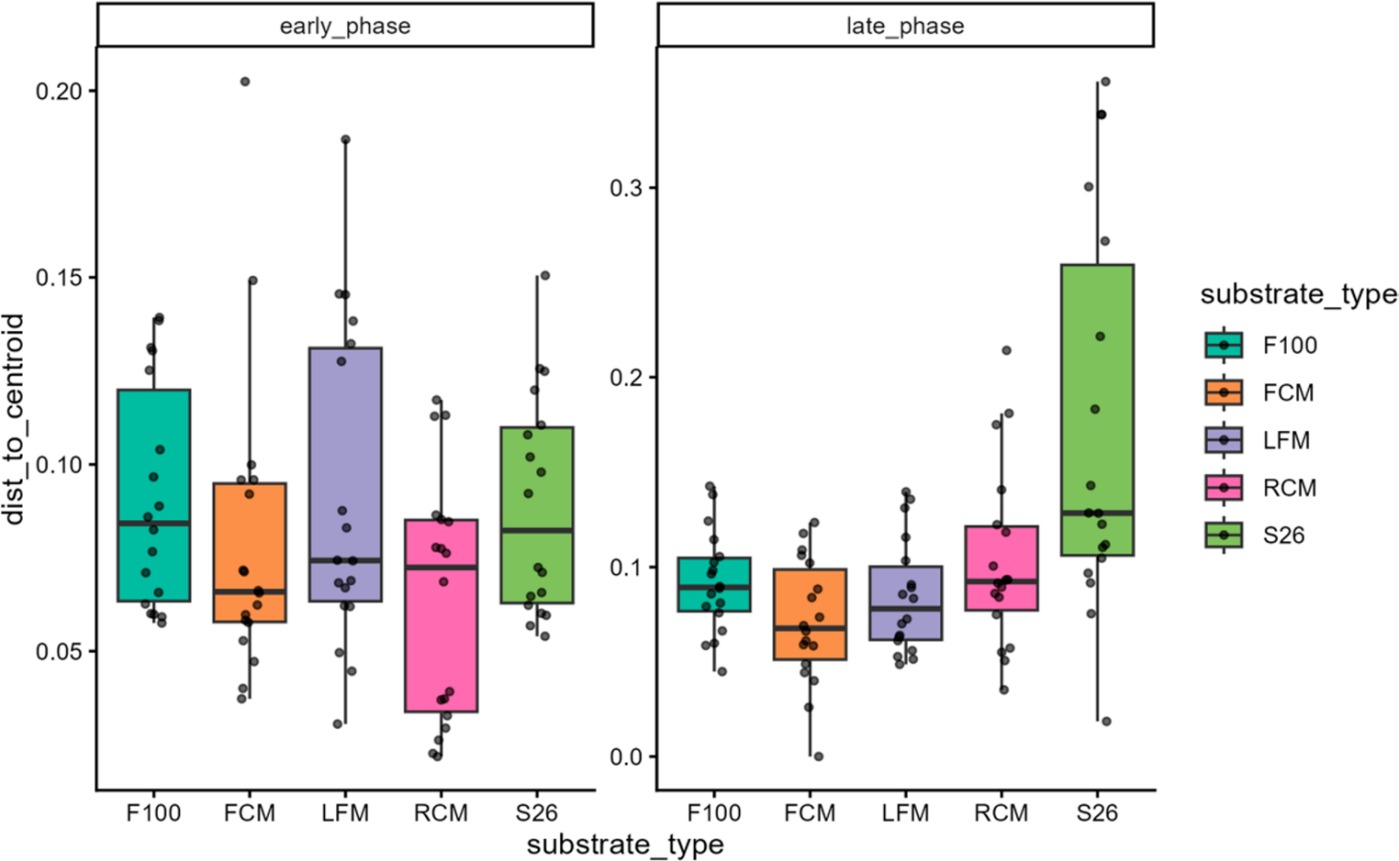
Dispersion analysis of microbial communities within sample groups following substrate treatments between propagation phases. The x-axis shows substrate treatments faceted into propagation phases, while the y-axis represents dispersion distance to centroids. The legend shows substrate groups in colors: F100: F100 infant formula (green), FCM: ultra-high temperature full-cream milk (cocoa brown), LFM: ultra-high temperature low-fat milk (purple yam), RCM: raw cow milk (deep cerise), S26: S26 infant formula (light aloe green)

**Fig. 6:**
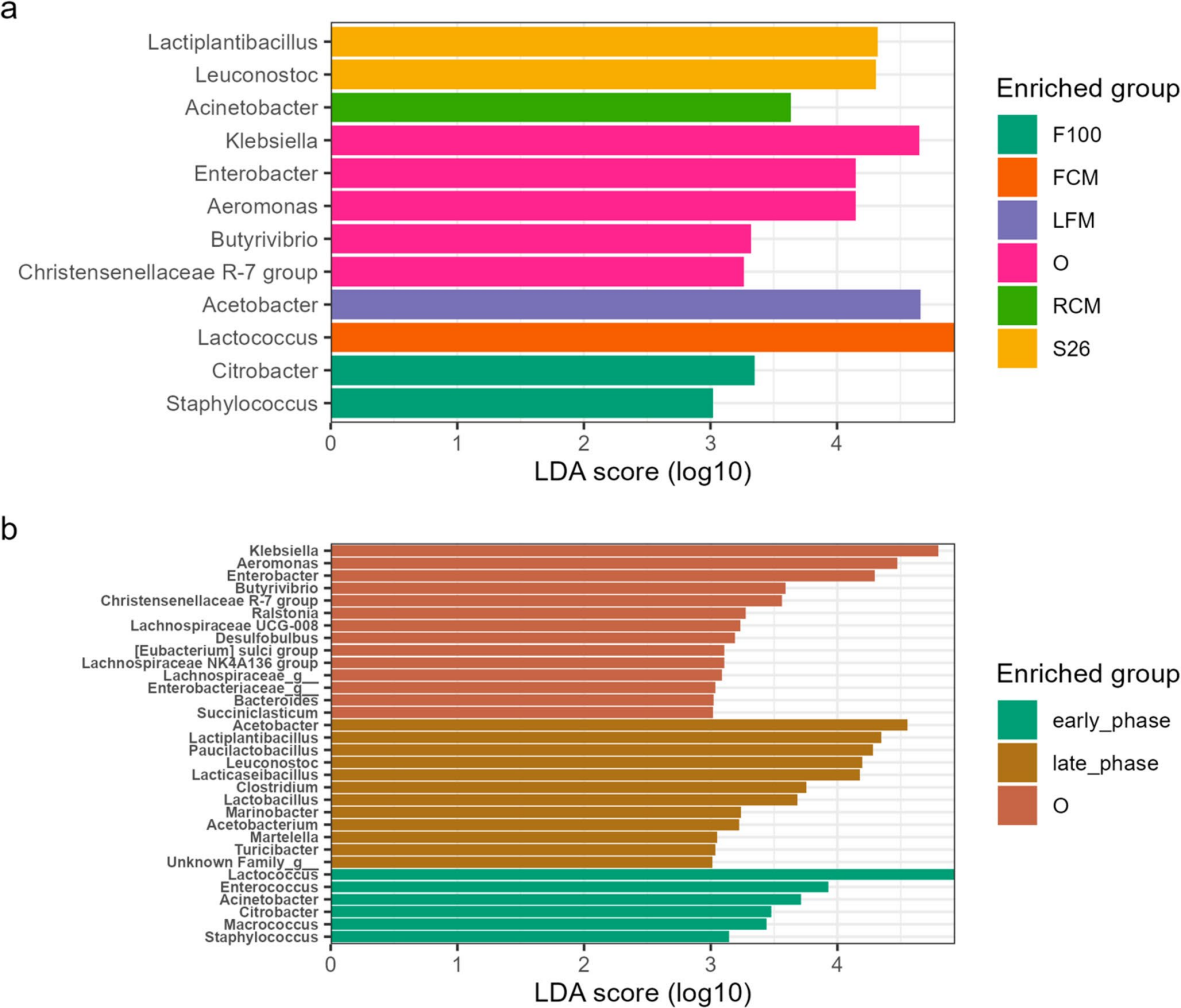
Linear discriminant analysis effect size (LEfSe) to identify differentially abundant microbial taxa following the propagation of mabisi in varied milk substrates over time. The x-axis shows the linear discriminant analysis (LDA) score (log 10 transformed effect size) of enriched taxonomic features according to substrate variation (Fig. 5a) and propagation phase (Fig. 5b). The y-axis lists the enriched taxa at genus level. The legends show the enriched groups distinguished by colors: substrates - F100: F100 infant formula (green), FCM: ultra-high temperature full-cream milk (cocoa brown), LFM: ultra-high temperature low-fat milk (purple yam), O: starting mabisi microbial community (deep cerise), RCM: raw cow milk (light aloe green), and S26: S26 infant formula (yellow); propagation phases – early_phase: early phase (dark teal), late_phase: late phase (brownish orange), and O: starting mabisi microbial community (warm terracotta)

Further analysis by linear discriminant effect size (LEfSe), focusing on LAB and AAB that are known for their key roles in dairy ecosystems, revealed that there were significant differences in the relative enrichment of the mabisi microbial community between substrate treatment and propagation phases. Notably, LDA analysis showed that members of the *Lactococcus* were significantly enriched in FCM (*LDA* = 5.00, *p* = 6.15e-03), *Acetobacter* were significantly enriched in LFM (*LDA* = 4.70, *p* = 1.52e-13), while *Lactiplantibacillus* (LDA = 4.34, *p* = 4.33e-03) and *Leuconostoc* (*LDA* = 4.32, *p* < 1.11e-09) were significantly enriched in S26 treatment (Fig. 5, Table S3). Similarly, members of the *Lactococcus* (*LDA* = 4.93, *p* = 7.55e-13) were significantly enriched during the early phase, while *Acetobacter* (*LDA* = 4.54, *p* = 2.08e-04), *Lactiplantibacillus* (*LDA* = 4.36, *p* = 3.70e-20), *Paucilactobacillus* (*LDA* = 4.30, *p* = 3.65e-26), *Leuconostoc* (*LDA* = 4.20, *p* = 3.34e-02), *Lacticaseibacillus* (*LDA* = 4.16, *p* = 6.36e-20) and *Lactobacillus* (*LDA* = 3.67, *p* = 2.53e-16) were significantly enriched during the late phases of propagation (Fig. 6, Table S4). However, only taxa other than those belonging to LAB and AAB were differentially enriched in farm site 1 (Fig. S4; Table S4). The differential enrichment outcome supports the observed community diversity changes.

## Microbial community-level functionality

To understand how the changes in the microbial community diversity influenced the community-level functionality following the repeated propagation of mabisi in varied substrates at different sites over time, volatile organic compounds (VOCs), pH, and consistency were analyzed as proxy indicators.

### Volatile organic compounds

The obtained VOCs belong to the chemical classes of aldehydes, esters, carboxylic acids, alcohols, and ketones (Table S5). The VOC profiles exhibited differences between samples analyzed before and after the propagation of mabisi across substrates and farm sites over time. Particularly, samples analyzed before propagation displayed VOCs that are associated with photo oxidation and undesirable flavors, including octanoic acid, nonanoic acid, ethyl-9 decenoate, and 1-octen-3-one, among others (Fig. S5). Therefore, these specific samples were removed from downstream analysis.

**Fig. 7:**
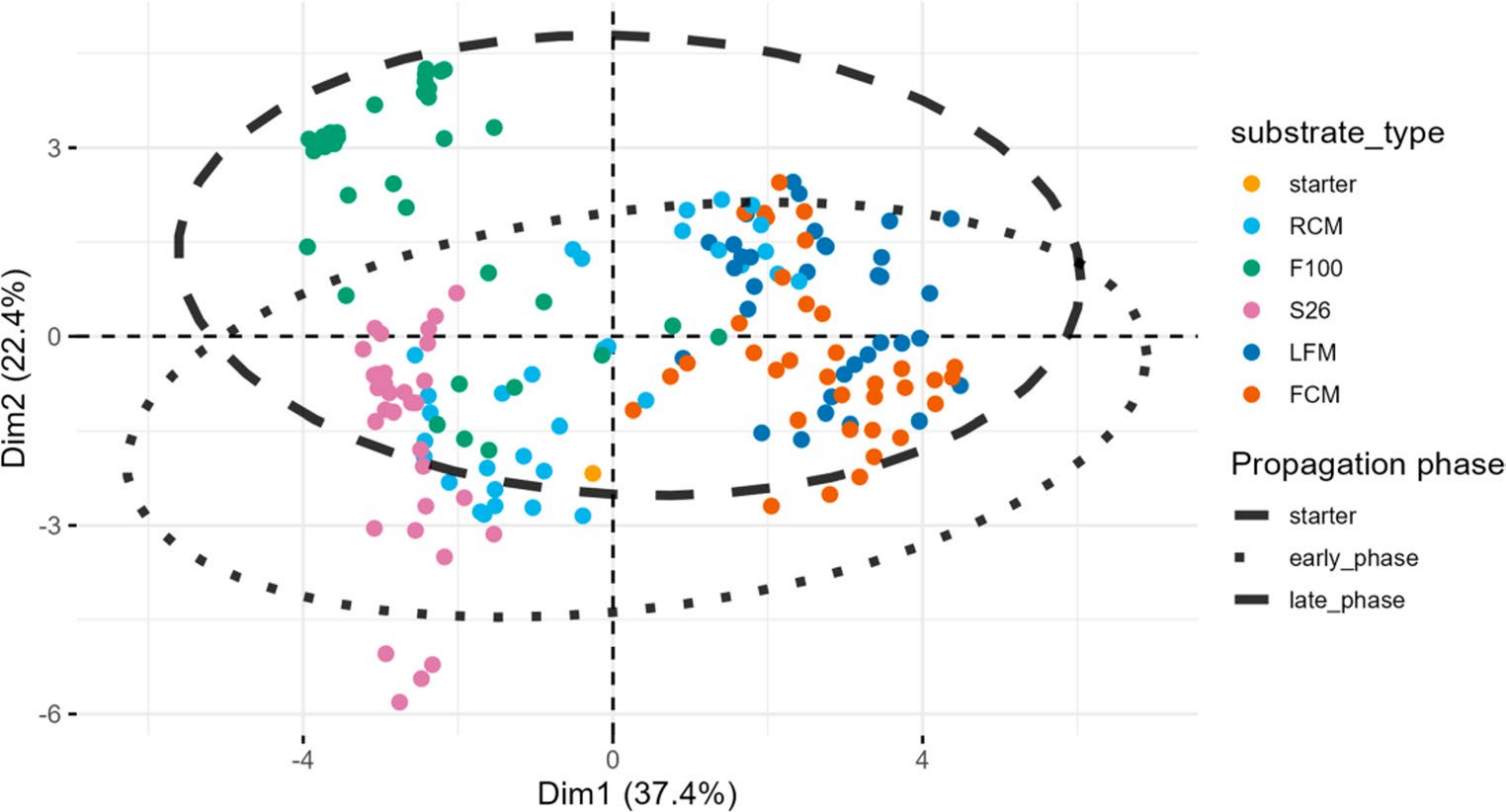
Volatile organic compounds from mabisi samples after propagation of mabisi microbiota in varied milk substrates over time. Each point represents an individual sample. Legends show substrates distinguished by color – starter: starting mabisi microbial community (orange), RCM: raw cow milk (sky blue), F100: F100 infant formula (green), S26: S26 infant formula (light pink), LFM: ultra-high temperature low-fat UHT (dark blue), and FCM: ultra-high temperature full-cream milk (dark orange). Propagation phases are represented by ellipses at 95% confidence interval – starter: starting mabisi microbial community (solid ellipse not formed due to inadequate data points), early_phase: early phase (dotted ellipse) and late_phase: late phase (dashed ellipse)

Further analysis of propagated samples through principal component analysis (PCA) and PERMANOVA revealed that VOCs were significantly driven by both the substrate treatment, which separated along the PC1 axis explaining 37.4% of the variation, and the propagation phase, which separated along the PC2 axis explaining 22.4% of the variation (*p* = 0.001) (Fig. 7; Table S6). Specifically, the VOCs in the F100 and S26 treatments, exhibited higher levels of Ethyl acetate, Hexanal, and 1 Butanol 3-Methyl, which separated from those belonging to LFM and FCM, which showed higher levels of 2-Heptanone, 2-Nonanone, Butanoic acid, and 2-Butanone, Octanoic acid, and Hexanoic acid, whereas, those from RCM appeared to contribute shared VOC from either group (Fig. S6). However, the farm site did not impact any significant separation of VOCs (*p* = 0.21) (Fig. S7; Table S6).

**Fig. 8:**
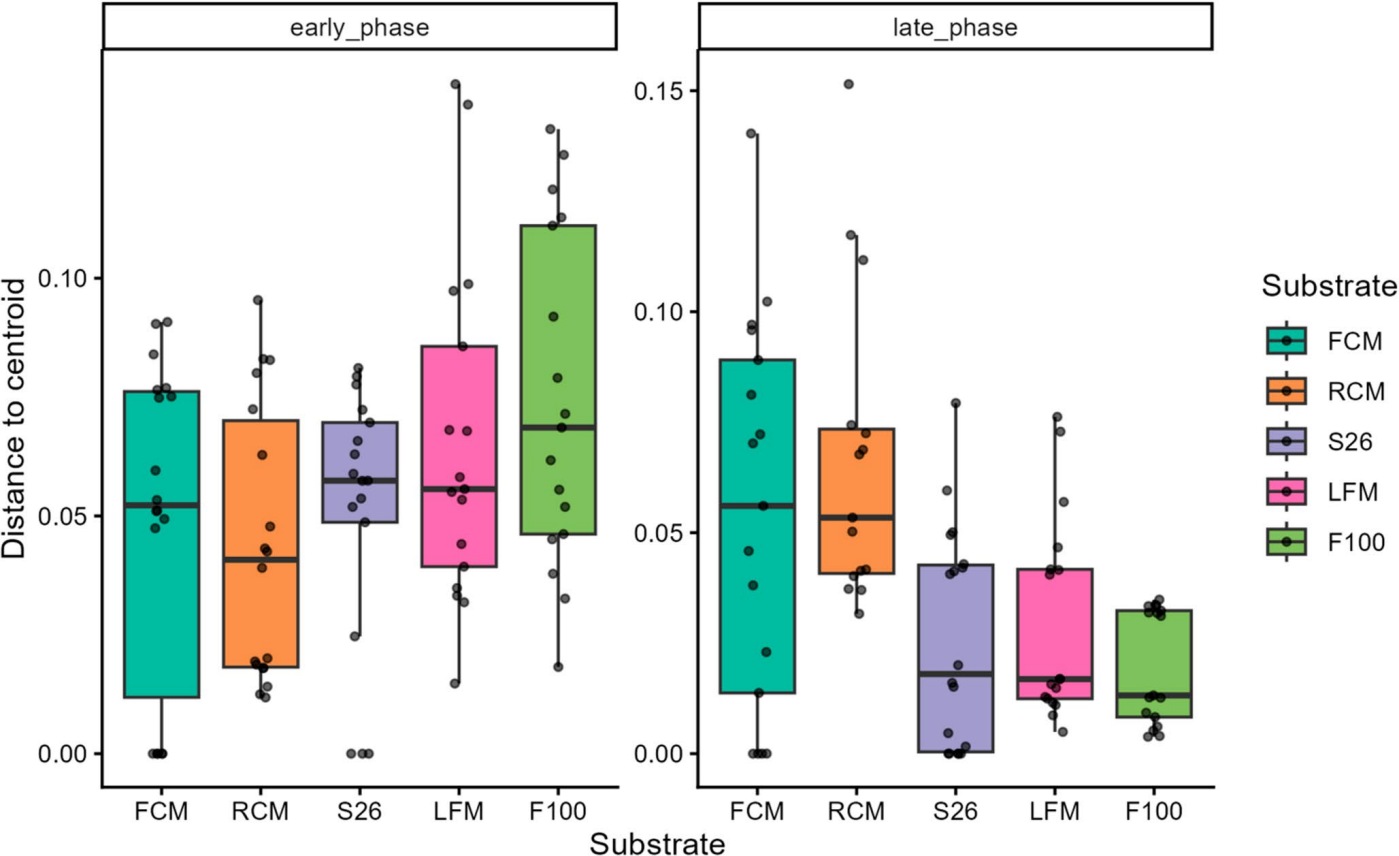
Dispersion analysis of volatile organic compounds within sample groups following substrate treatments between propagation phases. The x-axis shows substrate treatments faceted into propagation phases while the y-axis represents dispersion distance to centroids. The legend shows substrate groups in colors: FCM: ultra-high temperature full-cream milk (green), RCM: raw cow milk (cocoa brown), (S26: S26 infant formula (purple yam), LFM: ultra-high temperature low-fat milk (deep cerise), and F100: F100 infant formula (light aloe green)

Additionally, beta dispersion analysis was conducted to determine whether the variability in VOC profiles among substrates in both the early and late propagation phases was homogeneous. In the early phase, the analysis showed significant but marginal dispersion differences among substrates (F = 2.69, *p* = 0.036), which was mainly due to increased dispersion in F100 treatment (*p* = 0.049) (Fig. 8; Table S7). In the late phase, however, the variability in multivariate dispersion within substrate treatments was significantly different (F = 7.43, *p* = 0.001). In particular, RCM exhibited significantly higher dispersion compared to F100 (*p* < 0.001), S26 (*p* = 0.002), and LFM (*p* = 0.007) treatments but was comparable to FCM (*p* = 0.78) (Fig. 8; Table S7). This suggests that VOC profiles followed a heterogeneous divergence, which was more pronounced during the late propagation phase.

### pH and consistency

**Fig. 9:**
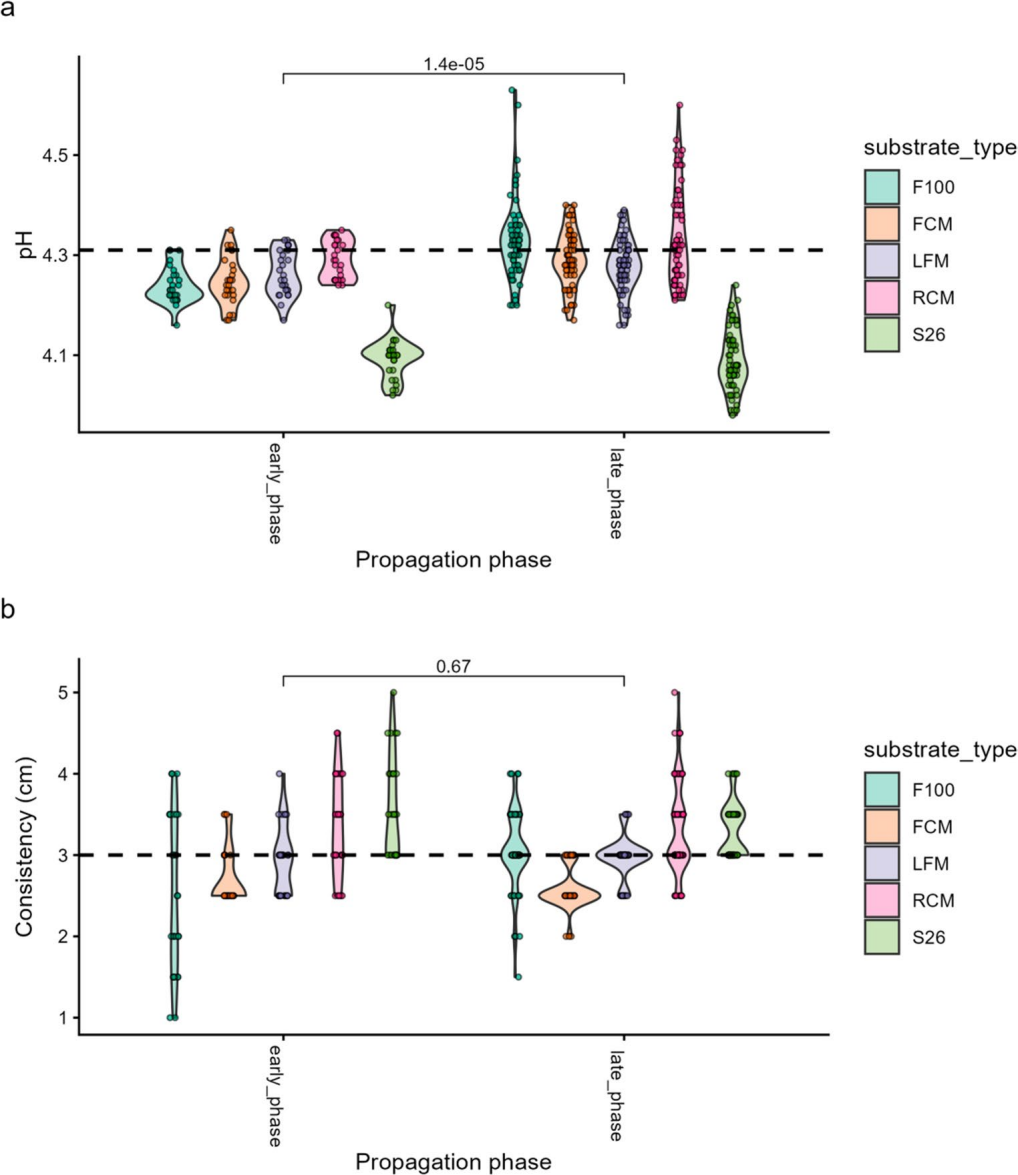
Violin plots of pH (Fig. 7a) and consistency (Fig. 7b) data following repeated propagation of mabisi in varied milk substrates over time. The x-axis shows the propagation phases: early phase (early_phase) and late phase (late_phase), while the y-axis shows the pH (Fig. 7a) and consistency (cm) (Fig. 7b) measurements. Dots represent individual sample data for each substrate at each propagation phase. Legends show the substrate treatment - F100: F100 infant formula (light green), FCM: ultra-heat temperature full-cream milk (light cocoa brown), LFM: ultra-high temperature low-fat milk (light purple yam), RCM: raw cow milk (light cerise), and S26: S26 infant formula (light aloe green). The dotted horizontal line represents the median value of pH (Fig. 7a) and consistency (Fig. 7b) for the reference substrate (RCM). The differences in median pH and consistency of substrates between propagation phases were analyzed by the Wilcox test, and numbers in the comparisons represent *p*-values

Following propagation, the pH dropped in all substrates to a range between 3.98 and 4.51 (Fig. 9a) from a range between 5.69 and 6.77 before propagation (Fig. S8a). When compared to the RCM reference substrate, the median pH for LFM (*p* = 0.006), FCM (*p* = 0.016), and S26 (*p* = 0.001) based substrate treatments was significantly lower, while that for F100 was not significantly different following the propagation process (*p* = 0.17) (Fig. 9a; Table S8). Further analysis revealed that the pH was significantly lower for propagated mabisi in the late propagation phase compared to the early phase (*p* = 1.40e-05) (Fig. S8a; Table S9), and was also significantly lower for samples from farm 2 compared to farm 1 (*p* < 0.001) and 3 (*p* < 0.001) (Fig. S8b; Table S10). Thus, the pH was driven by differences in the substrates, propagation, and farm sites.

In addition, the substrate treatments also influenced the consistency outcomes. The consistency ranged between 1.5 and 4.11 cm for all substrates following propagation of mabisi in varied substrates, with higher values observed in S26 and lower values observed in F100 treatments (Fig. 9b). Relative to the raw cow milk treatments, the median consistency was significantly lower for F100 (*p* < 0.001), FCM (*p* < 0.001), and LFM (*p* < 0.001) while that for S26-based treatments was not (*p* = 0.15) (Fig. 9b; Table S11). However, consistency was not significantly different between early and late propagation phases (*p* = 0.67) (Fig. 9b; Table S11) or between sites (*p* = 0.72) (Fig. S8c; Table S11). These outcomes suggest that substrate variation was the key determinant for driving consistency patterns.

## Discussion

In this study, we aimed to investigate how species sorting processes influence the assembly of natural microbial communities and maintain their functionality when exposed to novel environments across sites over time. Our findings partially supported our initial expectation, revealing a strong species sorting process evidenced by a substrate-driven divergence in microbial community composition linked to both the early and late propagation phases across sites. Although the community-level functionality remained largely repeatable relative to properties known for mabisi (Moonga et al. 2021), it also followed a substrate-driven pattern of divergence.

In our study, the propagated microbial communities remained diverse and dominated by community members belonging to lactic acid bacteria (LAB) and acetic acid bacteria (AAB), including *Lactococcus*, *Acetobacter*, *Leuconostoc*, *Lactiplantibacillus*, *Paucilactobacillus*, and *Lacticaseibacillus*. Accordingly, the spectra of LAB and AAB taxa observed here are consistent with previous reports in similar environments (Leale et al. 2023; Moonga et al. 2020), even from those found worldwide (Kochetkova et al. 2022; Liang et al. 2021). This suggests that these taxa are generalists and stable colonizers of mabisi niches, as they were able to repeatedly thrive in the novel environments imposed during our experiment. Microbial generalists are known to inherently possess metabolic flexibility to adapt to dynamic environments and, thus, have a wider niche breadth, as opposed to specialist taxa that require specific resources and conditions (Chen et al. 2021).

However, microbial communities undergo sorting, diverging into distinct clusters under the influence of environmental selection. This hypothesis has been tested in many studies, ranging from natural to controlled study models (Langenheder and Székely 2011; Székely et al. 2013; Zhang et al. 2020; Zhu et al. 2020). Therefore, our results resonate with this hypothesis, given that the propagated microbial community diverged in composition, linked to each substrate treatment, thereby reflecting the role of environmental selection processes in situ. Although the explicit mechanisms explaining how environmental selection shapes the assemblage of microbial communities are still unclear, we speculate that species sorting demonstrated in our findings could have been governed by physical-chemical and micronutrient variability between environments (Kochetkova et al. 2022; Zhang et al. 2020). The substrates used in our experiment were bovine-derived and homogeneous, yet they differed in pretreatment, pH, micronutrient content, and purpose (Kunda et al. 2015; Nyirenda et al. 2009; Park et al. 2012). As such, these abiotic factors likely exerted niche partitioning, driving differential adaptation of species, as observed in the consequent dissimilarity in community composition despite evolving from a shared starting community. For instance, the selective enrichment of *Lactobacillus* in S26 could be due to the high protein and mineral content in the substrate. This could serve as a source of a diverse range of peptides and essential amino acids, which promote the growth and stimulatory effects of *Lactobacillus* species (Bozoudi et al. 2015; Ma et al. 2016). Correspondingly, *Leuconostoc* was also enriched in S26 treatment. This is unsurprising, given that *Lactobacillus* and *Leuconostoc* are well known for their positive growth interaction in food fermentation (Yan et al. 2025). Although the explicit interplay remains unknown, this could partly be mediated by the exopolysaccharide produced by *Leuconostoc*, which mediates an oxygen stress remedial effect to *Lactobacillus* (Yan et al. 2018). Strikingly, *Acetobacter* was enriched in LFM, the substrate with lower nutrient profiles. This could be due to a quick depletion of the readily available lactose following the fermentation process. Thereafter, the byproducts could have then paved a niche opportunity to promote *Acetobacter*, which is known for its predilection for ethanol as a carbon source, particularly in co-culture interactions with *Lactobacilli* species (Chai et al. 2020). Conversely, *Lactococcus* dominated in a nutrient-rich FCM substrate, and its competitive urge could be attributed to its efficiency in utilizing readily available nutrients as well as its capability to synthesize bactericidal-like inhibiting substance (BLIS) against competitors (Bozoudi et al. 2015).

It is noteworthy that the temporal effect explained more community variation than substrate variation in our analysis. Our findings showed that the effect of species sorting continues over temporal scales, especially in the late propagation phase, relative to the early phase. A high nutrient availability has been shown to induce strong microbial interactions, resulting in altered chemical environments such as lowered pH that disfavor certain coexisting members over time (Ratzke et al. 2020). Consequently, this interaction predisposes other members to subsequent extinction and overall shifts in biodiversity. In our study, early colonizers such as *Lactococcus* dominated due to an optimal environment and their efficiency in utilizing available resources (Bozoudi et al. 2015), while members of the *Lactobacillus* dominated during the late fermentation phases, an indication of their ability to thrive in low pH. Their acid tolerance mechanisms are partly explained by decreasing their membrane fluidity through modulating cell membrane fatty acid composition (Huang et al. 2016). Moreover, our observed community composition remained divergent between substrates as indicated by differential analysis during the late propagation phase. This divergence pattern is concordant with previous observations (Johansen et al. 2019). In contrast, other studies indicate that species-sorting mechanisms are important during the initial phases of microbial assembly (Langenheder and Székely 2011; Székely et al. 2013). This temporal succession pattern is consistent with other dairy fermentation (Gapp et al. 2024), and even those for vinegar ecosystems (Zhu et al. 2018). The explicit mechanisms underpinning substrate-driven microbial diversity over extended temporal scales are largely unknown, although this could be attributed to evolution through the accumulation of genetic mutations, which confer microbial fitness gains or losses under selective pressure (Cubas-Cano et al. 2019; Feng et al. 2018; Lawrence et al. 2012; Naseeb et al. 2017). Thus, the evidence of a species-sorting process through successional patterns in microbial diversity over time in our study suggests that microbial communities stabilize into different structural organization over time, reminiscent of alternative stable states conceptualized on the ecological equilibrium landscape (Shaw et al. 2019).

In addition, other studies indicate that species-sorting mechanisms are influenced by location (Kochetkova et al. 2022; Zhang et al. 2020). This is congruent with our findings, which revealed that site differences exerted significant differences in the community diversity outcomes. We mimicked the locally adopted culture conditions at respective sites; site 1 involved an outdoor-based incubation on an elevated location, site 2 involved incubation in an enclosure on a wooden platform, while site 3 involved incubation in an enclosure but on the floor. These features could attract site-specific ecological differences, including in-house microbiota and temperature variations (Gobbetti et al. 2018; Quintana et al. 2020) that could contribute to the niche division and varied diversity trajectories during the species sorting process, hence the differences in the diversity patterns observed between the studied sites (Kochetkova et al. 2022). Additionally, we conducted a batch system and dispersal from exposure to in-house microbiota during experimental manipulations (Lee et al. 2013) could also have played a role during site-specific induced environmental selection. Conversely, the effect of farm sites explained a minimal and least variation for community divergency, as compared to temporal and substrate effects in our analysis. Therefore, this exemplifies that propagation of mabisi at different sites is robust and yields repeatable outcomes. This is further supported by the observed divergence between farm sites, which was determined as negligible differences at the level of bacterial guilds known for mabisi ecosystems (Leale et al. 2023; Moonga et al. 2020), as revealed by the differential abundance analysis. On the other hand, the three sites in our study comprised replicates of similar treatments and were located within a radius of two km, thereby assumed to experience similar weather conditions.

We further evaluated whether community-level functionality could mirror the trajectory of the observed microbial community diversity patterns. Similar to the functional properties known for mabisi (Moonga et al. 2021), we found repeatable patterns in the overall community-level functionality, as evidenced by the proxies of VOC production, and decline in pH, as well as the consistency. This suggests that the propagated microbial community displayed functional convergence in the context of mabisi (Groenenboom et al. 2022). However, the degree of specific functional capacities appeared to vary significantly. For instance, the VOC profile was significantly separated by substrate treatment along the PC1 axis, explaining 37.4% of the variation, and by propagation phases along the PC2 axis, accounting for 22.4% of the variation. This reflects that environmental-specific factors, such as variation of candidate micronutrient substrates for VOC production (Laëtitia et al. 2014; Smid and Kleerebezem 2014), could have instigated the microbial metabolic capacities to varying degrees for this trait. In addition, previous studies have shown that the dynamic changes in the species relative abundance correlate with shifts in VOC production (Dan et al. 2017; Walsh et al. 2016), which mirrors substrate-driven microbial community divergence observed in our study. Accordingly, our substrate treatments with enriched taxa such as *Acetobacter* and *Lactococcus* were associated with compounds including 3-hydroxy 2-butanone (acetoin) and 2-nonanone, *Leuconostoc* with octanoic acid, and *Lactobacillus* with benzoic acid but inversely associated with 2-heptanone, hexanoic acid ethyl ester, and octanoic acid ethyl ester, concordant with previous reports (Zheng et al. 2018, 2021). This is further in agreement with the reports that suggest that, despite the system’s functional repeatability, specific functions are impacted by the environmental selection during species sorting (Chen et al. 2021; Peter et al. 2011).

Conversely, the declining trend in pH, as in other fermented dairy systems (Sharma et al. 2023), appeared to be substrate-dependent between sites and over temporal scales in our study. This indicates that the observed community changes also performed the metabolic conversion of lactose to varying magnitudes. Our observations are consistent with a previous report, which revealed differences in pH reduction capacity due to microbial selection from temperature and processing effects (Moonga et al. 2021). Changes in pH further lead to strong niche partition and microbial interactions where better-adapted taxa are promoted while others are negatively selected (Mougi 2023; Ratzke et al. 2020), reflected by the differential abundance in microbial community composition between environments studied here. For instance, *Acetobacter*, *Lactiplantibacillus*,* Paucilactobacillus*,* Leuconostoc*, *Lacticaseibacillus*, and *Lactobacillus* appeared to increase in frequency over the late phase of propagation, particularly in the S26 treatment, which exhibited the lowest pH during that phase. Most of these taxa are known to constitute superior buffering capacities for low pH among LAB through a two-component system mediated by histidine protein kinase and the corresponding response regulator, collectively facilitating proton pump activity (Wang et al. 2018), including adjustment of cell wall fluidity (Huang et al. 2016), among other alleviative pathways for stress shock. Moreover, repeated propagation is reminiscent of adaptive evolution experiments, which have shown improved metabolic functional capacity towards desired functions following repeated exposure of microbial communities to selection (Konstantinidis et al. 2021; Rocchi et al. 2023). This underscores our observed high fermentation capacities reflected by the lower pH towards the late phase, compared to the early phase of propagation.

The coagulation of milk protein under acid conditions, as well as the production of exopolysaccharides, underlie the decline in consistency of fermented dairy systems (Lucey 2017; Nemati and Mozafarpour 2024). We found that consistency was driven by the substrate variation, whereas it remained indifferent over temporal and site scales. While it was evident that the protein fraction in the studied environments was different (Kunda et al. 2015; Nyirenda et al. 2009; Park et al. 2012), this could not explain the observed consistency patterns, given that the substrate with the highest protein fraction - S26, displayed an indifferent median consistency, whereas the F100, FCM, and LFM-based substrates exhibited significantly lower median consistency compared to RCM. This outcome, therefore, suggests that other unknown selective factors, or the substrate differentially enriched taxa, governed the consistency functional property through differential production of exopolysaccharides (Nemati and Mozafarpour 2024; Yang et al. 2023), whereas over temporal and spatial scales, this effect was negligible. Nevertheless, taken together, this could indicate that mabisi microbial community exhibits functional resilience when exposed to varied milk environments over time, regardless of site differences, and can be attributed to microbial community asynchrony, which promotes different taxa to perform overlapping functions over a temporal scale, thereby maintaining functional stability (Louca et al. 2016; Wagg et al. 2021).

Beyond the ecological implications highlighted above, our findings also hold biotechnological relevance. In particular, our study demonstrates that mabisi microbial communities can be harnessed for developing novel fermented food ingredients, including for purposes such as the fermentation of infant formula, where LAB may consequently confer probiotic benefits to infants (Radke et al. 2017). This further supports the feasibility and effectiveness of the current practice of its use in treating malnourished children in an in-house formulated F100 infant formula in low-income countries. Previous studies indicate that microbial communities can adapt and improve performance through adaptive evolutionary engineering (Konstantinidis et al. 2021; Rocchi et al. 2023). Accordingly, our study revealed that repeated propagation, reminiscent of adaptive evolution, represents a promising strategy to enhance such functional traits. Thus, future work should build on this approach by evaluating the efficacy of such novel fermented products in vivo, i.e., involving other spectra of human subjects, across age groups.

In our study, we were limited by the sole application of the 16 S rRNA V3-V4 amplicon sequencing for microbial profiling. This method is only capable of detecting bacterial and archaea taxa (Langille et al. 2013) and does not resolve species to strain levels of taxonomy. While strain level information would be interesting, the V3-V4 amplicon sequencing approach did allow for comparative analysis of selective response between communities, addressing our main research question. However, 16 S rDNA amplicon sequencing does not account for yeast. On the other hand, previous work showed that yeasts are not central members since they are not consistently present in mabisi, and when present, they are only represented by a single species (Moonga et al. 2020; Schoustra et al. 2013). This suggests that the bacteria (LAB and AAB) community underlie species sorting dynamics in mabisi niches. Further, our study approach could not account for genetic mutations (Feng et al. 2018) over the experimental evolution time scale, which could have complemented the observed trajectory of community diversity and its function. Nevertheless, by revealing dissimilarity in community diversity and repeatable community-level functionality in propagated novel environments across sites over time, this study has revealed ecological insights into how species sorting sustains a natural microbial community and functionality *in situ.* A set of starting communities should be heat-treated as a control to ensure loss of viability and inoculated at the same biomass as the test starter, in replicated sterile substrates, and propagated similarly to the main experiment (Bourrie et al. 2022). Thereafter, the microbial community composition and fermentation kinetics should be determined and compared. This approach could account for potential background fermentation, thereby helping to infer our observations beyond association to the mechanistic causality of ecological selection and functionality of the Mabisi starter across substrates.

In conclusion, our study has shown that, following exposure to novel environments, a natural microbial community from a common starting community diverges in composition, albeit through attaining alternative stable states on an ecological equilibrium landscape. The repeatability in metabolic profiles across environmental treatments also reflects functional redundancy. Our study not only serves to inform fundamental ecological insights on the microbial community responses to environmental changes, but also provides a basis for harnessing a natural mabisi microbial community to be diversified for biotechnological applications to enhance novel fermented food ingredients.

## Supplementary information

Below is the link to the electronic supplementary material.

Supplementary File 1 (DOC X 1.01 MB)

Supplementary File 2 (DOCX 19.0 KB)

Supplementary File 3 (DOCX 13.9 KB)

Supplementary File 4 (DOCX 17.7 KB)

Supplementary File 5 (DOCX 26.8 KB)

Supplementary File 6 (DOCX 15.8 KB)

Supplementary File 7 (DOCX 20.5 KB)

Supplementary File 8 (DOCX 23.3 KB)

Supplementary File 9 (DOCX 16.6 KB)

Supplementary File 10 (DOCX 13.7 KB)

Supplementary File 11 (DOCX 14.5 KB)

Supplementary File 12 (DOCX 17.3 KB)

## Data Availability

The datasets generated during the current study are available online in the 4TU.ResearchData repository: [https://doi.org/10.4121/82fa6380-c40c-4c40-b5ec-933b0253e6ce](https:/doi.org/10.4121/82fa6380-c40c-4c40-b5ec-933b0253e6ce).
