# Supplementary material for: Species sorting shapes the divergence of a traditional fermented dairy-derived bacterial community with repeatable functionality during propagation with alternative substrates
Source: World J Microbiol Biotechnol. 2026 Apr 28;42(5):243. doi: 10.1007/s11274-026-04830-3 (PMC13124831; doi:10.1007/s11274-026-04830-3)
Supplement: Supplementary file 1 — (DOC X 1.01 MB) [file 11274_2026_4830_MOESM1_ESM.docx]

**SUPPLEMENTARY INFORMATION**

**Composition and diversity of microbial communities**

[Please insert Fig. S1 here]


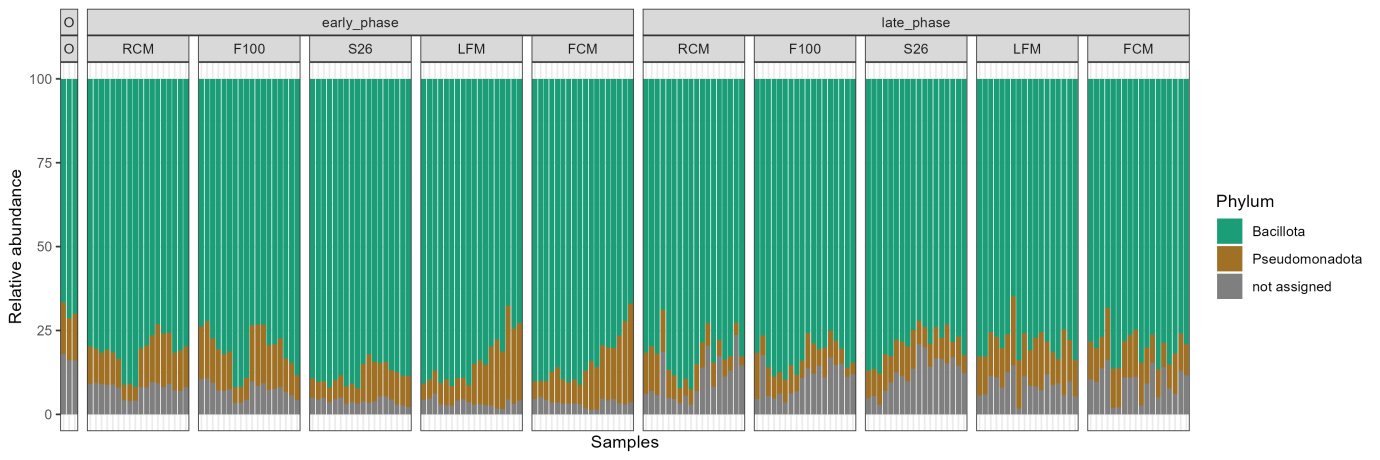


**Fig. S1** Spectra of mabisi microbial communities at the phylum level of classification. A shared mabisi microbial community was propagated in different substrates over time. Facets represent starting mabisi microbial community (O), the early phase of propagation (early_phase), and the late phase of propagation (late_phase). Each substrate treatment is represented by starting mabisi microbial community (O), raw cow milk (RCM), F100 infant formula (F100), S26 infant formula (S26), ultra-high temperature low-fat milk (LFM), and ultra-high temperature full-cream milk (FCM). The y-axis shows the relative abundance (%) of each taxon per sample, while the x-axis represents samples. The legend shows the list of the most abundant phyla: Bacillota (dark teal), Pseudomonadota (brownish orange), and not assigned – referring to the amplicon sequence variants not annotated to any phyla in the SILVA database (gray).

[Please insert Fig. S2 here]


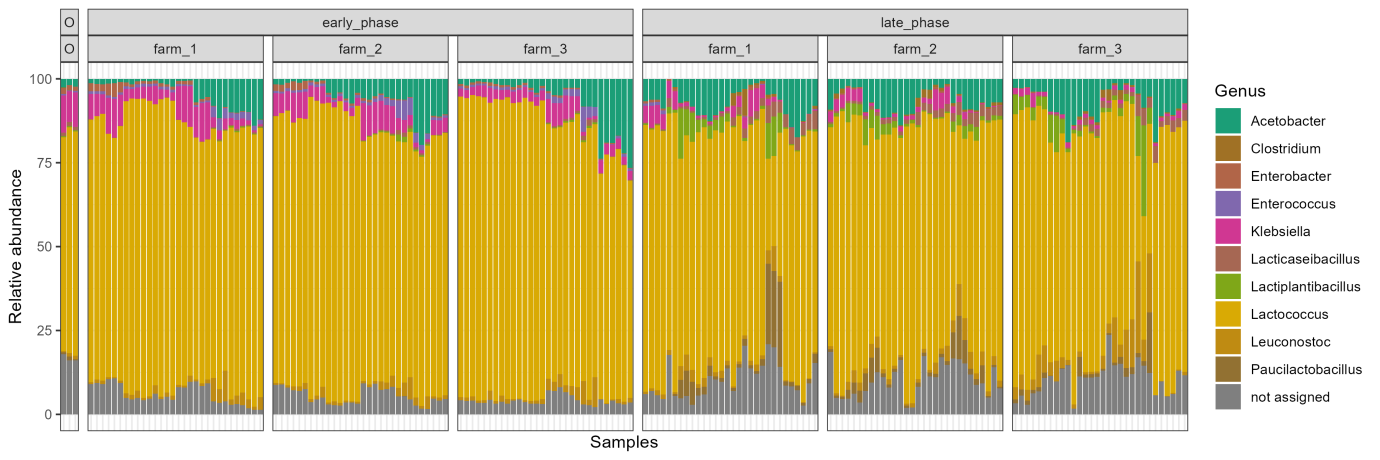


**Fig. S2** Distribution of mabisi microbial community following propagation in varied substrates over time at different farm sites. The y-axis represents the relative abundance (%) of taxa in each sample. The x-axis represents samples, faceted into propagation phases - starting mabisi microbial community (O), early phase (early_phase), and late phase (late_phase), while the farm sites are represented by farm site 1 (farm_1), farm site 2 (farm_2), and farm site 3 (farm_3). The legend shows the list of the most abundant genera; *Acetobacter* (dark teal), *Clostridium* (brownish orange), *Enterobacter* (warm terracotta), *Enterococcus* (soft lavender purple), *Klebsiella* (magenta), *Lacticaseibacillus* (mutated brick red), *Lactiplantibacillus* (bright olive green), *Lactococcus* (golden yellow), *Leuconostoc* (dark mustard yellow), *Paucilactobacillus* (earthly brown), and unassigned - referring to ASVs that could not be annotated in the silva database (medium gray)

[Please insert Fig. S3 here]

**Fig. S3** Alpha diversity of mabisi microbial communities at different farm sites over time. Chao1 (richness) and Shannon (incorporating both richness and evenness) diversity metrics were applied to assess microbial community alpha diversity following propagation of a shared starting mabisi microbial community in varied milk substrates. The y-axis represents the diversity indices (Chao1 and Shannon), while the x-axis represents propagation phases: starting mabisi microbial community (O), early phase (early_phase), and late phase (late_phase). Box plots display the median, interquartile range, whiskers, and outliers. The legend shows the varied farm sites: farm site 1 (farm_1, medium aquamarine), farm site 2 (farm_2, coral), farm site 3 (farm_3, dark pastel blue), and starting mabisi microbial community (O, middle purple)

*Differential abundance analysis*

[Please insert Fig. S4 here]


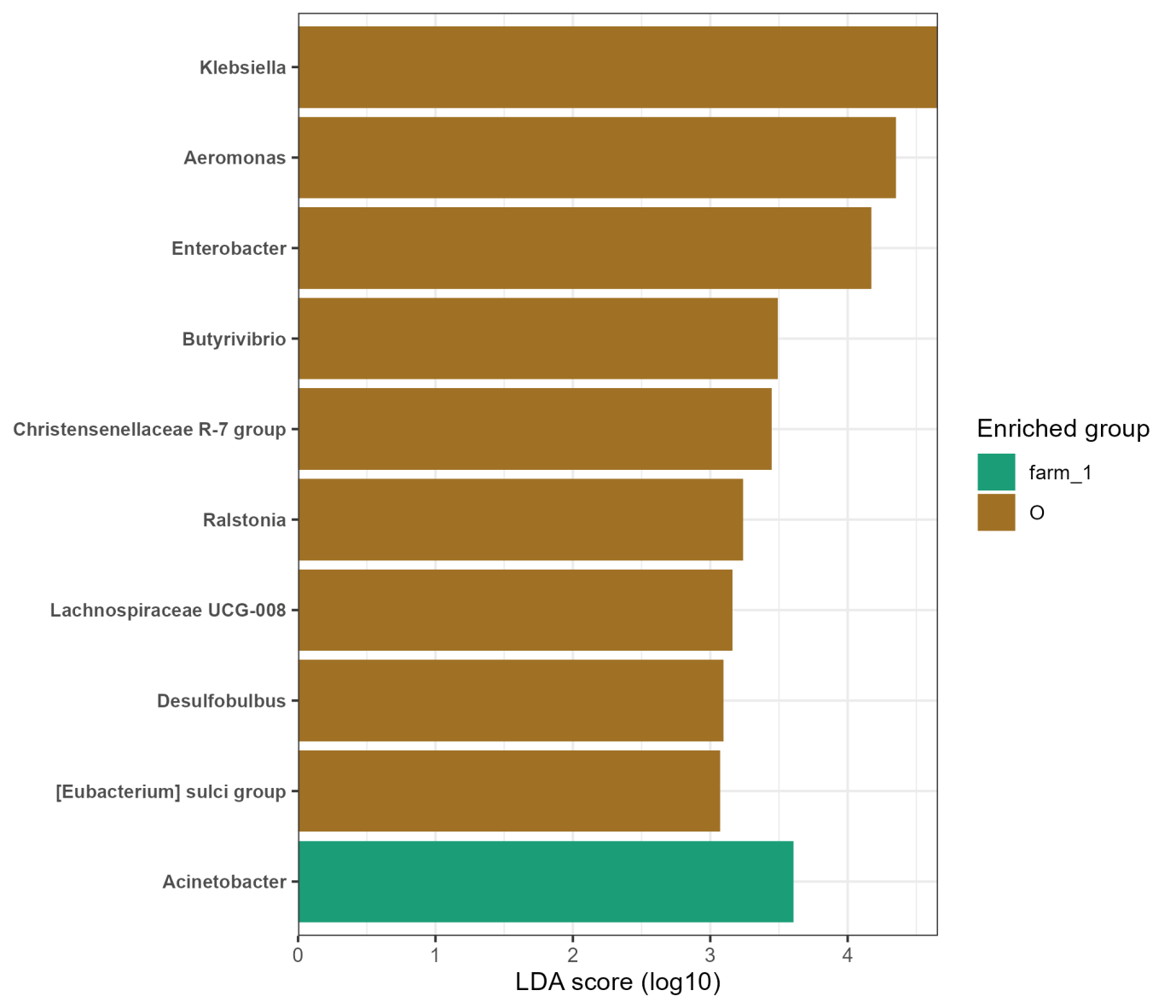


**Fig. S4** Linear discriminant analysis effect size (LEfSe) showing differentially abundant microbial taxa by farm site. The x-axis shows the linear discriminant analysis (LDA) score (log 10 transformed effect size) of enriched taxonomic features according to farm site. The y-axis lists the enriched taxa at the genus level. The legends show the enriched groups: farm 1 (farm_1, dark teal) and the starting mabisi microbial community (O, brownish orange)

**Microbial community-level functionality**

*Volatile Organic Compounds*

[Please insert Fig. S5 here]


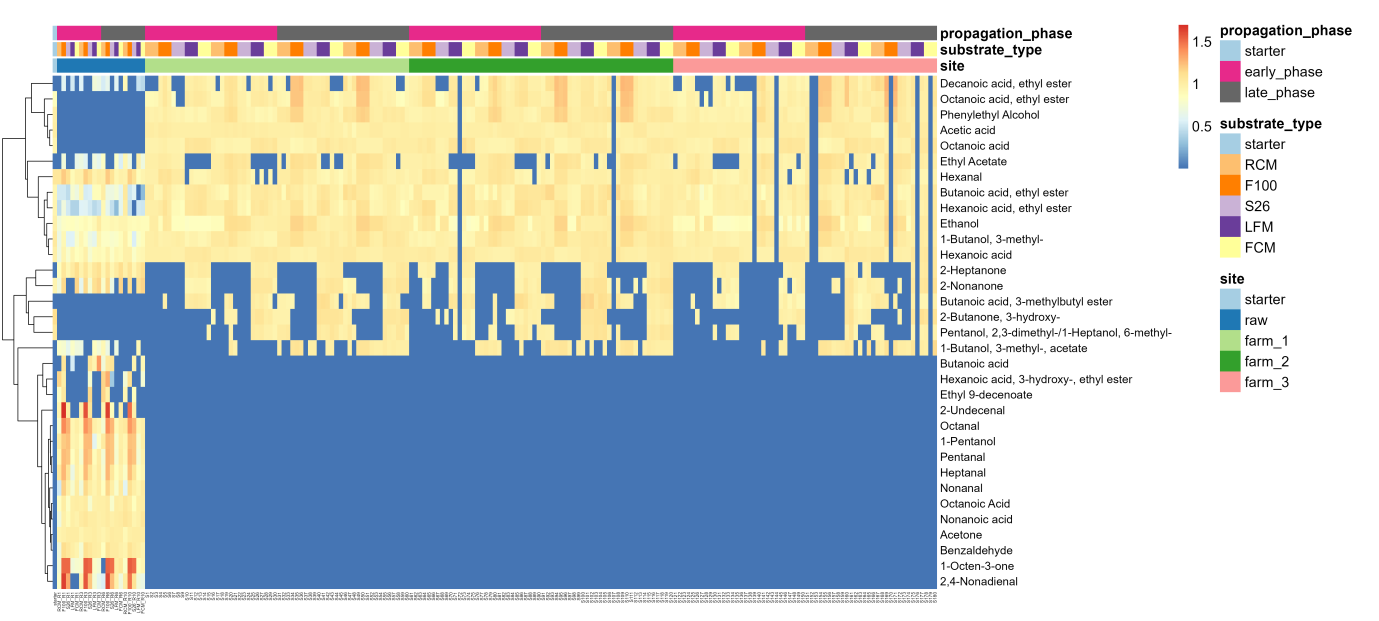


**Fig. S5** Heatmap of volatile organic compounds (VOCs) detected before and after propagation of mabisi microbial communities in varied milk substrates over time. The x-axis represents the samples, and the y-axis lists the VOC names. Scaling by color indicates normalized VOC abundance after log transformation and median scaling by each compound, with red denoting high intensity, and blue representing low intensity. Legend denote three grouping factors: (i) propagation phase - starter: starting mabisi microbial community (light blue), early_phase: early phase (pink), and late_phase: late phase (gray); (ii) substrate type - starter: starting mabisi microbial community (light blue), RCM: raw cow milk (light orange), F100: F100 infant formula (orange), S26: S26 infant formula (light purple), LFM: ultra-high temperature low-fat milk (purple), and FCM: ultra-high temperature full-cream milk (light yellow); and (iii) farm site - starter: starting mabisi microbial community (light blue), raw: substrates before propagation at each farm (blue), farm_1: farm site 1(light green), farm_2: farm site 2 (green) and farm_3: farm site 3(light red)

[Please insert Fig. S6 here]


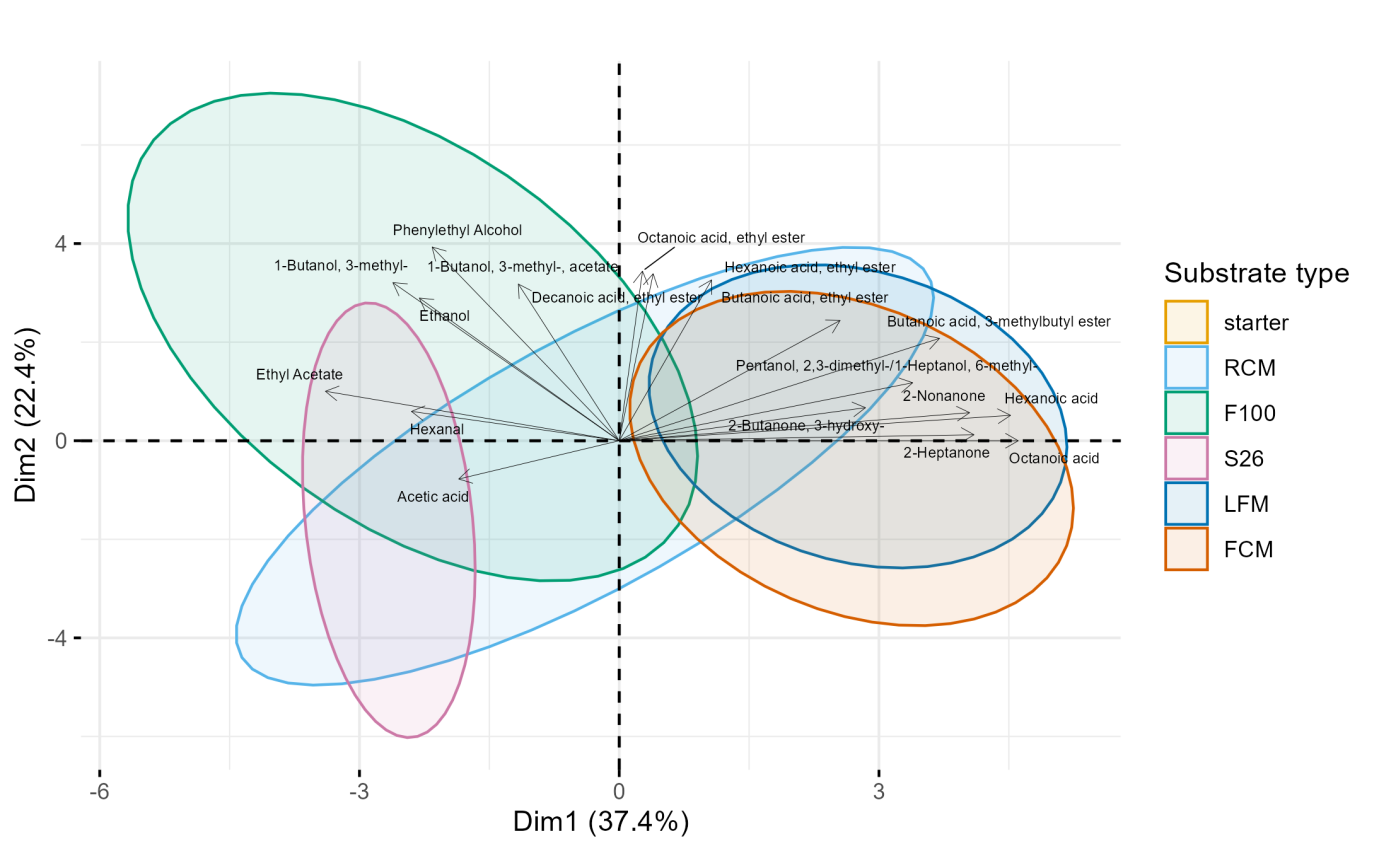


**Fig. S6** Clusters of the most contributing (top 18) volatile organic compounds from mabisi samples after propagation with varied milk substrates. Legends show substrate types including – starter: starting mabisi microbial community (orange), RCM: raw cow milk (sky blue), F100: F100 infant formula (green), S26: S26 infant formula (light pink), LFM: ultra-high temperature low-fat milk (dark blue), and FCM: ultra-high temperature full-cream milk (dark orange). Ellipses represent a 95% confidence interval of VOC distribution for each substrate group (with the starter lacking an ellipse due to inadequate data points)

[Please insert Fig. S7 here]


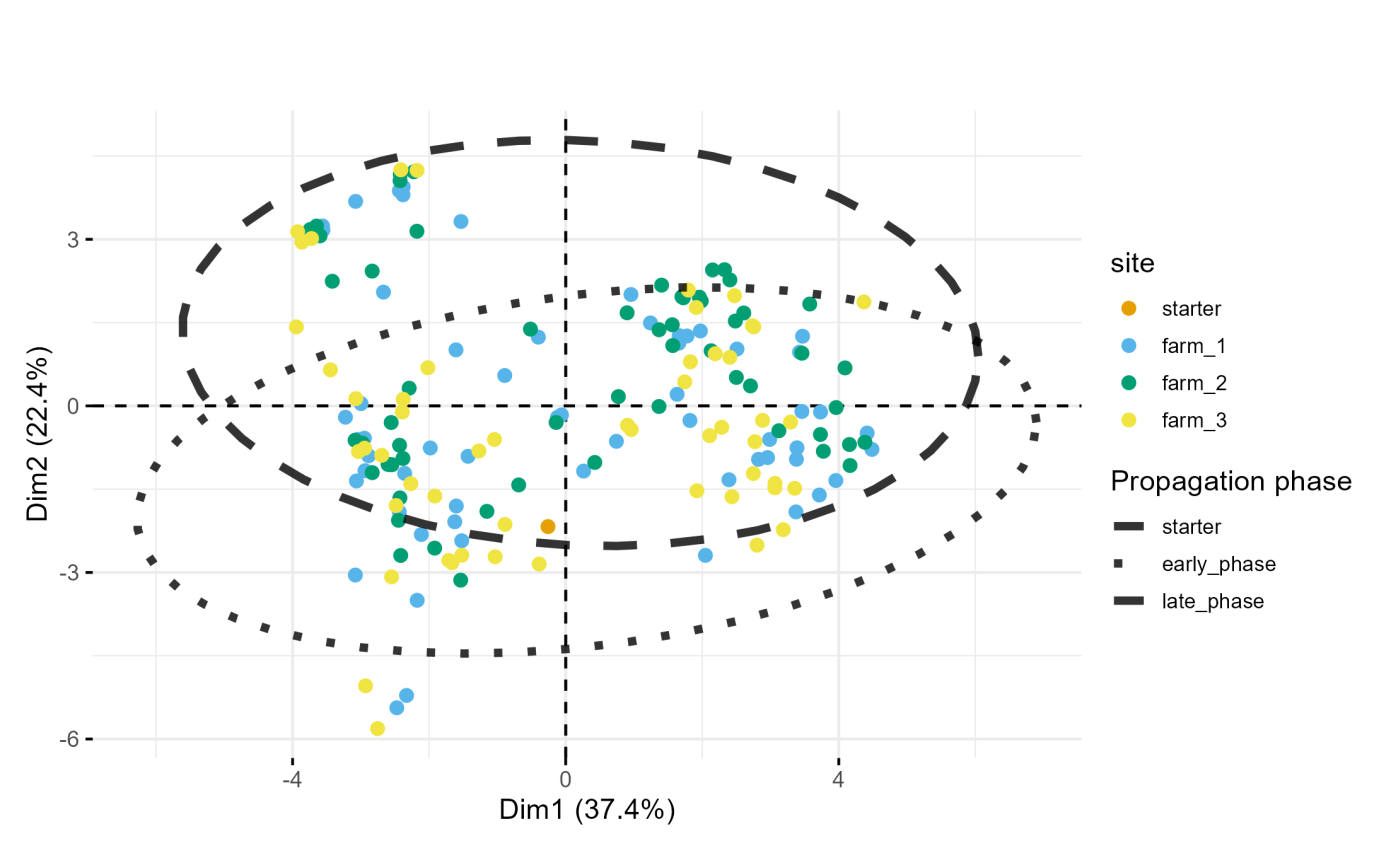


**Fig. S7** Clustering of volatile organic compounds from mabisi samples after propagation of mabisi microbiota in varied milk substrates at different farm sites. The points in the graph represent each sample. Farm sites – starter: starting mabisi microbial community (orange), farm_1: farm site 1 (sky blue), farm_2: farm site 2 (green), farm_3: farm site 3 (yellow). Propagation phases include – starter: starting community (solid ellipse not formed due to inadequate data points, orange); early_phase: early phase (dotted ellipse) and late_phase: late phase (dashed ellipse). Ellipses represent a 95% confidence interval of VOC distribution for each farm site

*pH and consistency*

[Please insert Fig. S8 here]


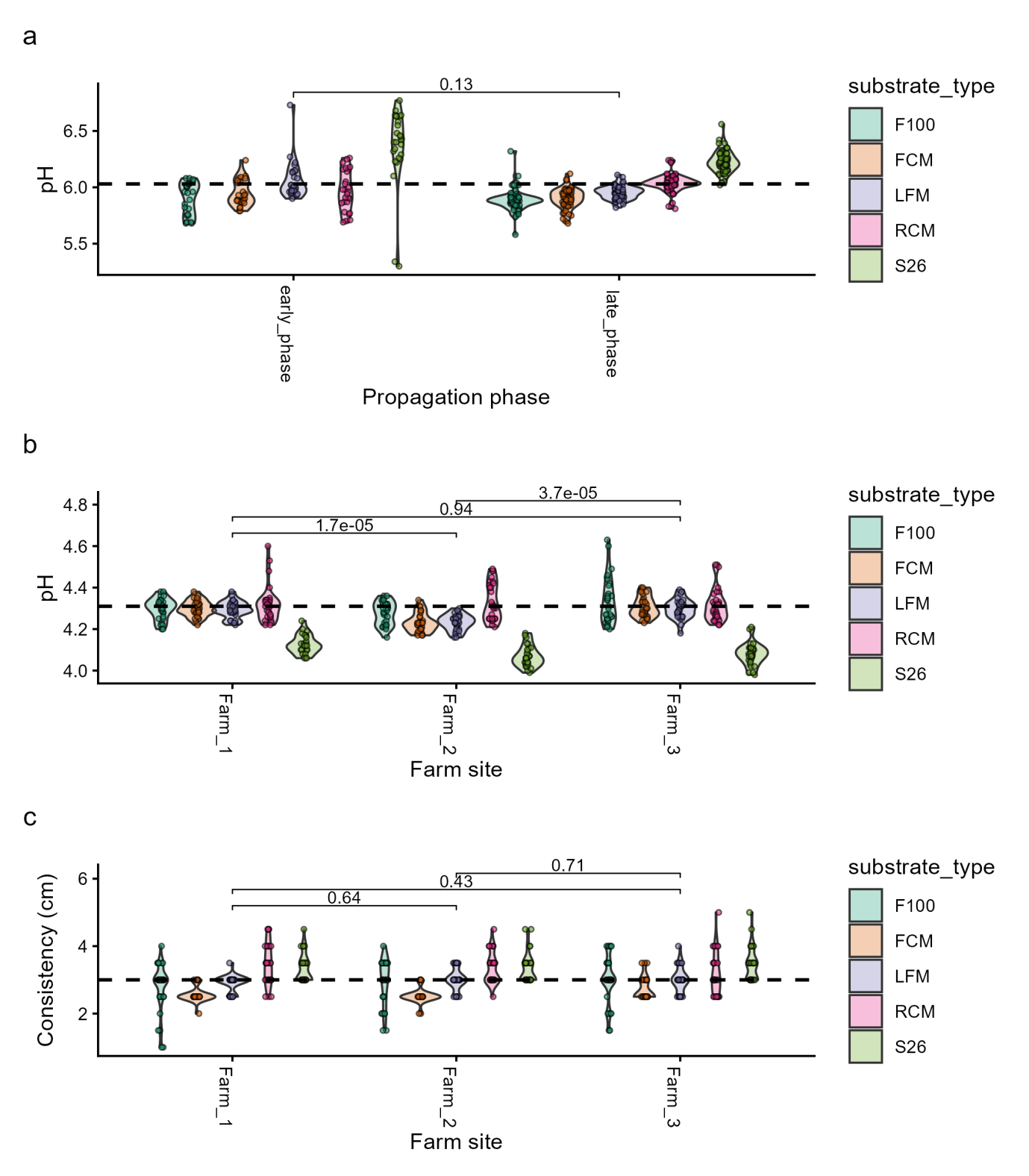


**Fig. S8** Violin plots of pH before propagation (Fig. S8a), after propagation (Fig. S8b) of mabisi and its consistency (Fig. S8c) at different farm sites. Dots represent data from individual samples for each substrate treatment. The y-axis shows the range of the pH (Fig. S8a & Fig. S8b), as well as that of the consistency (Fig. S8c), while the x-axis shows farm sites (Fig. S8b & Fig. S8c): farm 1, farm 2, and farm 3. Legends show the substrate treatment - F100: F100 infant formula (light green), FCM: ultra-heat temperature full-cream milk (light cocoa brown), LFM: ultra-high temperature low-fat milk (light purple yam), RCM: raw cow milk (light cerise), and S26: S26 infant formula (light aloe green). The pairwise analysis of the differences in median pH and consistency of substrates between the propagation phases and farm sites was analyzed by the Wilcox test, with numbers in the comparisons representing statistical *p*-values
