# Supplementary material for: Species sorting shapes the divergence of a traditional fermented dairy-derived bacterial community with repeatable functionality during propagation with alternative substrates
Source: World J Microbiol Biotechnol. 2026 Apr 28;42(5):243. doi: 10.1007/s11274-026-04830-3 (PMC13124831; doi:10.1007/s11274-026-04830-3)
Supplement: Supplementary file 2 — (DOCX 19.0 KB) [file 11274_2026_4830_MOESM2_ESM.docx]

**Table S1** Alpha diversity of mabisi microbial communities assessed using Chao1 (richness) and Shannon (richness and evenness) indices. The pairwise Wilcoxon rank-sum test was applied to evaluate differences across substrate treatments, propagation phases, and farm sites, with *p*-values adjusted using the Benjamin-Hochberg method

| **Variable** | **Group 1** | **Group 2** | **Chao1**  **Adj. *p*-value** | **Shannon**  **Adj. *p*-value** |
| --- | --- | --- | --- | --- |
| **Substrate variation** | FCM | F100 | 0.115 | 0.001 * |
|  | LFM | F100 | 0.058 | 0.080 |
|  | O | F100 | 0.058 | 0.005* |
|  | RCM | F100 | 0.64 | 0.299 |
|  | S26 | F100 | 0.572 | 0.057 |
|  | LFM | FCM | 0.562 | 0.349 |
|  | O | FCM | 0.058 | 0.001* |
|  | RCM | FCM | 0.297 | 0.018* |
|  | S26 | FCM | 0.093 | < 0.001* |
|  | O | LFM | 0.058 | 0.001* |
|  | RCM | LFM | 0.115 | 0.220 |
|  | S26 | LFM | 0.058 | 0.001* |
|  | RCM | O | 0.058 | 0.002* |
|  | S26 | O | 0.115 | 0.299 |
|  | S26 | RCM | 0.458 | 0.004* |
| **Propagation phase** | Late phase | Early phase | 0.068 | 0.072 |
|  | O | Early phase | 0.007* | 0.014* |
|  | O | Late phase | 0.068 | 0.043* |
| **Farm site** | Farm 2 | Farm 1 | 0.978 | 0.994 |
|  | Farm 3 | Farm 1 | 0.243 | 0.375 |
|  | O | Farm 1 | 0.056 | 0.039* |
|  | Farm 3 | Farm 2 | 0.243 | 0.375 |
|  | O | Farm 2 | 0.066 | 0.037* |
|  | O | Farm 3 | 0.056 | 0.037* |

**Note:**

- ‘*’ represents statistically significant, while no esthetics represent a non-statistically significant result.
- Substrates are represented by - RCM: raw cow milk, F100: F100 infant formula, S26: S26 infant formula, LFM: ultra-high temperature low-fat milk, and FCM: ultra-high temperature full-cream milk, whereas O: refers to the starting mabisi microbial community for all variables.
