# Supplementary material for: Species sorting shapes the divergence of a traditional fermented dairy-derived bacterial community with repeatable functionality during propagation with alternative substrates
Source: World J Microbiol Biotechnol. 2026 Apr 28;42(5):243. doi: 10.1007/s11274-026-04830-3 (PMC13124831; doi:10.1007/s11274-026-04830-3)
Supplement: Supplementary file 3 — (DOCX 13.9 KB) [file 11274_2026_4830_MOESM3_ESM.docx]

**Table S2** Dissimilarity in microbial community composition across sample groups based on substrate variation, propagation phases and farm site differences. The analysis was conducted using a permutational multivariate analysis of variance (ANOSIM), with free permutation (n = 999)

| **Parameter** | **Anosim statistic R** | **P value** |
| --- | --- | --- |
| Substrate variation | 0.1635 | 0.001* |
| Propagation phase | 0.4008 | 0.001* |
| Farm site | 0.02767 | 0.014* |

**Note:** Significance codes: ‘*’ represents significant, and no asterisk represents a non-significant result.
