# Supplementary material for: Species sorting shapes the divergence of a traditional fermented dairy-derived bacterial community with repeatable functionality during propagation with alternative substrates
Source: World J Microbiol Biotechnol. 2026 Apr 28;42(5):243. doi: 10.1007/s11274-026-04830-3 (PMC13124831; doi:10.1007/s11274-026-04830-3)
Supplement: Supplementary file 4 — (DOCX 17.7 KB) [file 11274_2026_4830_MOESM4_ESM.docx]

**Table S3** Beta dispersion analysis was applied to assess the dispersion of microbial communities within samples following substrate treatments between propagation phases. A permutation test (using 999 permutations) was conducted, and *p*-values adjusted using Tukey's HSD method

| **Early propagation phase** | | | | | | |
| --- | --- | --- | --- | --- | --- | --- |
| **Permutation test:**  Response | **DF** | **Sum Sq** | **Mean Sq** | **F statistic** | **N. Perm** | **Pr (>F)** |
| Substrate groups | 4 | 0.01003 | 0.0025075 | 1.9558 | 999 | 0.111 |
| Residuals | 85 | 0.10898 | 0.0012821 |  |  |  |
| **Late propagation phase** | | | | | | |
| **Permutation test:**  Response | **DF** | **Sum Sq** | **Mean Sq** | **F statistic** | **N. Perm** | **Pr (>F)** |
| Substrate groups | 4 | 0.11900 | 0.0297492 | 9.4416 | 999 | 0.001* |
| Residuals | 85 | 0.26782 | 0.0031509 |  |  |  |

**Note:**

- ‘*’ represents statistically significant, while no esthetics represent a non-statistically significant result.
