# Supplementary material for: Species sorting shapes the divergence of a traditional fermented dairy-derived bacterial community with repeatable functionality during propagation with alternative substrates
Source: World J Microbiol Biotechnol. 2026 Apr 28;42(5):243. doi: 10.1007/s11274-026-04830-3 (PMC13124831; doi:10.1007/s11274-026-04830-3)
Supplement: Supplementary file 5 — (DOCX 26.8 KB) [file 11274_2026_4830_MOESM5_ESM.docx]

**Table S4** Differential abundance of microbial communities across substrates, propagation phases and sites. The analysis was conducted by linear discriminant effect size (LEfSe) applying an LDA cut-off of 3.

| **Variable group** | **Marker** | **Feature** | **Enriched group** | **Linear discriminant analysis score** | **p-value** | **Adjusted p-value** |
| --- | --- | --- | --- | --- | --- | --- |
| **Substrate variation** | marker1 | *Citrobacter* | F100 | 3.343751 | 1.046e-03* | 1.046e-03* |
|  | marker2 | *Staphylococcus* | F100 | 3.025378 | 8.673e-07* | 8.673e-07* |
|  | marker3 | *Lactococcus* | FCM | 4.998115 | 6.150e-03* | 6.150e-03* |
|  | marker4 | *Acetobacter* | LFM | 4.650600 | 1.517e-13* | 1.517e-13* |
|  | marker5 | *Klebsiella* | O | 4.640921 | 1.176e-12* | 1.176e-12* |
|  | marker6 | *Enterobacter* | O | 4.149835 | 1.498e-09* | 1.498e-09* |
|  | marker7 | *Aeromonas* | O | 4.137861 | 4.400-03* | 4.400e-03* |
|  | marker8 | *Butyrivibrio* | O | 3.337407 | 2.920e-02* | 2.920e-02* |
|  | marker9 | *Christensenellaceae_R7 group* | O | 3.288024 | 3.639e-02* | 3.639e-02* |
|  | marker10 | *Acinetobacter* | RCM | 3.615725 | 6.940e-05* | 6.940e-05* |
|  | marker11 | *Lactiplantibacillus* | S26 | 4.344082 | 4.328e-03* | 4.328e-03* |
|  | marker12 | *Leuconostoc* | S26 | 4.322887 | 1.113e-09* | 1.113e-09* |
| **Propagation phase** | marker1 | *Lactococcus* | early_phase | 4.928977 | 7.548e-13* | 7.548e-13* |
|  | marker2 | *Enterococcus* | early_phase | 3.926898 | 1.739e-26* | 1.739e-26* |
|  | marker3 | *Acinetobacter* | early_phase | 3.729863 | 6.363e-15* | 6.363e-15* |
|  | marker4 | *Citrobacter* | early_phase | 3.492008 | 2.990e-12* | 2.990e-12* |
|  | marker5 | *Macrococcus* | early_phase | 3.449128 | 2.610e-22* | 2.610e-22* |
|  | marker6 | *Staphylococcus* | early_phase | 3.156242 | 8.857e-05* | 8.857e-05* |
|  | marker7 | *Acetobacter* | late_phase | 4.541038 | 2.080e-04* | 2.080e-04* |
|  | marker8 | *Lactiplantibacillus* | late_phase | 4.359025 | 3.701e-20* | 3.701e-20* |
|  | marker9 | *Paucilactobacillus* | late_phase | 4.300808 | 3.653e-26* | 3.653e-26* |
|  | marker10 | *Leuconostoc* | late_phase | 4.204485 | 3.338e-02* | 3.338e-02* |
|  | marker11 | *Lacticaseibacillus* | late_phase | 4.159312 | 6.361e-20* | 6.361e-20* |
|  | marker12 | *Clostridium* | late_phase | 3.730698 | 1.011e-27* | 1.011e-27* |
|  | marker13 | *Lactobacillus* | late_phase | 3.674086 | 2.534e-16* | 2.534e-16* |
|  | marker14 | *Acetobacterium* | late_phase | 3.230790 | 5.826e-09* | 5.826e-09* |
|  | marker15 | *Marinobacter* | late_phase | 3.200596 | 3.838e-07* | 3.838e-07* |
|  | marker16 | *Martelella* | late_phase | 3.104545 | 1.461e-06* | 1.461e-06* |
|  | marker17 | *Turicibacter* | late_phase | 3.022426 | 1.213e-08* | 1.213e-08* |
|  | marker18 | *Klebsiella* | O | 4.798854 | 3.327e-13* | 3.327e-13* |
|  | marker19 | *Aeromonas* | O | 4.474495 | 4.067e-03* | 4.067e-03* |
|  | marker20 | *Enterobacter* | O | 4.293117 | 9.223e-16* | 9.223e-16* |
|  | marker21 | *Butyrivibrio* | O | 3.590815 | 2.606e-10* | 2.606e-10* |
|  | marker22 | *Christensenellaceae R-7 group* | O | 3.561722 | 2.048e-17* | 2.048e-17* |
|  | marker23 | *Ralstonia* | O | 3.281826 | 8.959e-10* | 8.959e-10* |
|  | marker24 | *Lachnospiraceae UCG-008* | O | 3.235014 | 2.368e-10* | 2.368e-10* |
|  | marker25 | *Desulfobulbus* | O | 3.193606 | 1.554e-14* | 1.554e-14* |
|  | marker26 | *Eubacterium sulci group* | O | 3.114361 | 4.034e-10* | 4.034e-10* |
|  | marker27 | *Lachnospiraceae NK4A136 group* | O | 3.085041 | 2.657e-06* | 2.657e-06* |
|  | marker28 | *Lachnospiraceae* | O | 3.084323 | 7.196e-11* | 7.196e-11* |
|  | marker29 | *Enterobacteriaceae* | O | 3.034241 | 1.513e-05* | 1.513e-05* |
|  | marker30 | *Bacteroides* | O | 3.032954 | 1.159e-08* | 1.159e-08* |
|  | marker31 | *Succiniclasticum* | O | 3.023009 | 3.879e-10* | 3.879e-10* |
| **Farm site** | marker1 | *Acinetobacter* | farm_1 | 3.594774 | 1.412e-02* | 1.412e-02* |
|  | marker2 | *Klebsiella* | O | 4.665998 | 7.339e-03* | 7.338709e-03* |
|  | marker3 | *Aeromonas* | O | 4.358014 | 6.706e-03* | 6.706e-03* |
|  | marker4 | *Enterobacter* | O | 4.156926 | 5.615e-03* | 5.615e-03* |
|  | marker5 | *Butyrivibrio* | O | 3.450271 | 3.556e-06* | 3.556e-06* |
|  | marker6 | *Christensenellaceae R-7 group* | O | 3.446018 | 9.215e-03* | 9.215e-03* |
|  | marker7 | *Ralstonia* | O | 3.237125 | 6.967e-03* | 6.967e-03* |
|  | marker8 | *Lachnospiraceae UCG-008* | O | 3.131719 | 1.479e-06* | 1.479e-06* |
|  | marker9 | *Eubacterium sulci group* | O | 3.050074 | 2.687e-06* | 2.687e-06* |
|  | marker10 | *Desulfobulbus* | O | 3.049411 | 6.426e-04* | 6.426e-04* |
|  | marker11 | *Lachnospiraceae* | O | 3.000355 | 1.155e-03* | 1.155e-03* |

**Note:** Differentially enriched substrate groups are represented by RCM: raw cow milk, F100: F100 infant formula, S26: S26 infant formula, LFM: ultra-high temperature low-fat milk, and FCM: ultra-high temperature full-cream milk. Differentially enriched propagation phase groups are represented by O: starting mabisi microbial community, early_phase: early propagation phase, and late_phase: late propagation phase. Differentially enriched Farm site groups are represented by farm_1: farm site 1 and O: stating mabisi microbial community. ‘*’ represents statistically significant results (*p* < 0.05). The *p*-values are rounded off to 3 decimal places.
