# Supplementary material for: Species sorting shapes the divergence of a traditional fermented dairy-derived bacterial community with repeatable functionality during propagation with alternative substrates
Source: World J Microbiol Biotechnol. 2026 Apr 28;42(5):243. doi: 10.1007/s11274-026-04830-3 (PMC13124831; doi:10.1007/s11274-026-04830-3)
Supplement: Supplementary file 6 — (DOCX 15.8 KB) [file 11274_2026_4830_MOESM6_ESM.docx]

**Table S5** The profile of volatile organic compounds (VOC) before and after repeated propagation of mabisi microbial community in varied milk substrates over time

|  | **Esters** | **Alcohols** | **Carboxylic acids** | **Ketones** | **Aldehydes** |
| --- | --- | --- | --- | --- | --- |
| Before propagation | Ethyl acetate | Ethanol | Nonanoic acid | 2-Heptanone | Hexanal |
|  | Butanoic acid, ethy ester | 1-Butanol, 3-methyl- | Hexanoic acid | Acetone | Heptanal |
|  | Hexanoic acid, ethyl ester | 1-Pentanol | Butanoic acid | 1-Octen-3-one | Nonanal |
|  | Decanoic acid, ethyl ester |  | Octanoic acid | 2-Nonanone | Benzaldehyde |
|  | Hexanoic acid, 3-hydroxy-, ethyl ester |  |  |  | 2,4-Nonadienal |
|  | Ethyl 9-decenoate |  |  |  | Pentanal |
|  | 1-Butanol, 3-methyl-, acetate |  |  |  | Octanal |
| After propagation | Ethy acetate | Ethanol | Acetic acid | 2-Heptanone | Hexanal |
|  | Butanoic acid, ethy ester | 1-Butanol, 3-methyl- | Hexanoic acid | 2-Butanone, 3-hydroxy- |  |
|  | 1-Butanol, 3-methyl-, acetate | Pentanol, 2,3-dimethyl-/1-Heptanol, 6-methyl- | Octanoic acid | 2-Nonanone |  |
|  | Hexanoic acid, ethyl ester | Phenylethyl alcohol |  |  |  |
|  | Butanoic acid, 3-methylbutyl ester |  |  |  |  |
|  | Octanoic acid, ethyl ester |  |  |  |  |
|  | Decanoic acid, ethyl ester |  |  |  |  |
