# Supplementary material for: Species sorting shapes the divergence of a traditional fermented dairy-derived bacterial community with repeatable functionality during propagation with alternative substrates
Source: World J Microbiol Biotechnol. 2026 Apr 28;42(5):243. doi: 10.1007/s11274-026-04830-3 (PMC13124831; doi:10.1007/s11274-026-04830-3)
Supplement: Supplementary file 7 — (DOCX 20.5 KB) [file 11274_2026_4830_MOESM7_ESM.docx]

**Table S6** Variation in volatile organic compounds following the repeated propagation of mabisi in varied substrates at different farm sites over time. The analysis was conducted by a permutational multivariate analysis of variance through Bray-Curtis distance metrics, with a free permutation (n = 999). The p-values for multiple testing were corrected by the Benjamin Hochberg method.

| **Parameter** | | **Df** | **SumOfSqs** | **R^2^** | **F** | ***p*-value** | ***p*- value**  **adjusted** |
| --- | --- | --- | --- | --- | --- | --- | --- |
| **Substrate variation** | | 5 | 1.7750 | 0.62665 | 55.725 | 0.001* | - |
| Residual | | 166 | 1.0575 | 0.37335 |  |  |  |
| Total | | 171 | 2.8325 | 1.00000 |  |  |  |
| ***Pairwise analysis:*** | |  |  |  |  |  |  |
| **Substrate group 1** | **Substrate group 2** |  |  |  |  |  |  |
| Starter | RCM | 1 | 0.03009611 | 0.07865297 | 2.731756 | 0.029* | 0.435 |
| Starter | F100 | 1 | 0.06507900 | 0.28617819 | 13.230025 | 0.029* | 0.435 |
| Starter | S26 | 1 | 0.04450728 | 0.21398061 | 9.255931 | 0.025* | 0.405 |
| Starter | LFM | 1 | 0.03270530 | 0.15741160 | 6.165030 | 0.023* | 0.375 |
| Starter | FCM | 1 | 0.02394310 | 0.10499712 | 3.988705 | 0.029* | 0.345 |
| RCM | F100 | 1 | 0.10972165 | 0.17566761 | 13.851688 | 0.001* | 0.015* |
| RCM | S26 | 1 | 0.09347474 | 0.15335987 | 11.955199 | 0.001* | 0.015* |
| RCM | LFM | 1 | 0.47015505 | 0.47120715 | 57.921482 | 0.001* | 0.015* |
| RCM | FCM | 1 | 0.47550045 | 0.46069321 | 56.379323 | 0.001* | 0.015* |
| F100 | S26 | 1 | 0.12179263 | 0.27209518 | 25.045001 | 0.001* | 0.015* |
| F100 | LFM | 1 | 0.58452859 | 0.63403336 | 114.344305 | 0.001* | 0.015* |
| F100 | FCM | 1 | 0.73197814 | 0.66640457 | 133.842081 | 0.001* | 0.015* |
| S26 | LFM | 1 | 0.85556142 | 0.71648161 | 169.316241 | 0.001* | 0.015* |
| S26 | FCM | 1 | 0.89018489 | 0.70775021 | 164.677668 | 0.001* | 0.015* |
| LFM | FCM | 1 | 0.01223147 | 0.03125150 | 2.161398 | 0.118 | 1.000 |
| **Propagation phase** | | 2 | 0.32002 | 0.11298 | 10.763 | 0.001* | - |
| Residual | | 169 | 2.51251 | 0.88702 |  |  |  |
| Total | | 171 | 2.83253 | 1.00000 |  |  |  |
| *Pairwise analysis:* | |  |  |  |  |  |  |
| Propagation group 1 | Propagation group 2 |  |  |  |  |  |  |
| Starter | Early phase | 1 | 0.02564651 | 0.01687570 | 1.476222 | 0.171 | 0.513 |
| Starter | Late phase | 1 | 0.03762413 | 0.03562723 | 3.066304 | 0.027* | 0.081 |
| Early phase | Late phase | 1 | 0.28998848 | 0.10347499 | 19.505617 | 0.001* | 0.003* |
| **Farm site** | | 3 | 0.06822 | 0.02408 | 1.382 | 0.212 | - |
| Residual | | 168 | 2.76431 | 0.97592 |  |  |  |
| Total | | 171 | 2.83253 | 1.00000 |  |  |  |

**Note:**

- ‘*’ represents statistical significance, and no esthetics represent a non-statistically significant result.
- Substrate types include raw cow milk (RCM), F100 infant formula (F100), S26 infant formula (S26), ultra-high temperature low-fat milk (LFM), and ultra-high temperature full-cream milk (FCM), while ‘starter’ represents the starting mabisi microbial community for all variables.
