# Supplementary material for: Species sorting shapes the divergence of a traditional fermented dairy-derived bacterial community with repeatable functionality during propagation with alternative substrates
Source: World J Microbiol Biotechnol. 2026 Apr 28;42(5):243. doi: 10.1007/s11274-026-04830-3 (PMC13124831; doi:10.1007/s11274-026-04830-3)
Supplement: Supplementary file 8 — (DOCX 23.3 KB) [file 11274_2026_4830_MOESM8_ESM.docx]

**Table S7** Beta dispersion analysis was applied to assess the variance of volatile organic acid compounds in samples following substrate treatments between propagation phases. A permutation test (using 999 permutations) was conducted, and *p*-values adjusted using Tukey's HSD method

| **Early propagation phase** | | | | | | |
| --- | --- | --- | --- | --- | --- | --- |
| **Permutation test:**  Response | **DF** | **Sum Sq** | **Mean Sq** | **F statistic** | **N. Perm** | **Pr (>F)** |
| Groups | 4 | 0.011371 | 0.0028428 | 2.6899 | 999 | 0.036* |
| Residuals | 82 | 0.086661 | 0.0010569 |  |  |  |
| **Pairwise test**  **(Tukey’s HSD)** | **Substrate group** | |  |  |  |  |
|  | **Group 1** | **Group2** | **difference** | **Lower 95% CI** | **Upper 95% CI** | **Adj. *p*-value** |
|  | F100 | RCM | 0.030684784 | 1.638993e-05 | 0.061353177 | 0.049* |
|  | S26 | RCM | 0.007241573 | -2.342682e-02 | 0.037909967 | 0.965 |
|  | LFM | RCM | 0.022180174 | -8.488220e-03 | 0.052848568 | 0.267 |
|  | FCM | RCM | 0.005482816 | -2.474428e-02 | 0.035709915 | 0.987 |
|  | S26 | F100 | -0.023443210 | -5.454664e-02 | 0.007660218 | 0.229 |
|  | LFM | F100 | -0.008504610 | -3.960804e-02 | 0.022598818 | 0.9408 |
|  | FCM | F100 | -0.025201967 | -5.587036e-02 | 0.005466426 | 0.158 |
|  | LFM | S26 | 0.014938601 | -1.616483e-02 | 0.046042029 | 0.667 |
|  | FCM | S26 | -0.001758757 | -3.242715e-02 | 0.028909637 | 0.999 |
|  | FCM | LFM | -0.016697358 | -4.736575e-02 | 0.013971036 | 0.553 |
| **Late propagation phase** | | | | | | |
| **Permutation test:**  Response | **DF** | **Sum Sq** | **Mean Sq** | **F statistic** | **N. Perm** | **Pr (>F)** |
| Groups | 4 | 0.026220 | 0.0065551 | 7.4251 | 999 | 0.001* |
| Residuals | 79 | 0.069744 | 0.0008828 |  |  |  |
| **Pairwise test**  **(Tukey’s HSD)** | **Substrate group** | |  |  |  |  |
|  | **Group 1** | **Group2** | **difference** | **Lower 95% CI** | **Upper 95% CI** | **Adj. *p*-value** |
|  | F100 | RCM | -0.046567096 | -0.0759520265 | -0.01718217 | <0.001* |
|  | S26 | RCM | -0.040733070 | -0.0697328602 | -0.01173328 | 0.002* |
|  | LFM | RCM | -0.036902571 | -0.0662875011 | -0.00751764 | 0.007* |
|  | FCM | RCM | -0.012032156 | -0.0414170867 | 0.01735277 | 0.783 |
|  | S26 | F100 | 0.005834026 | -0.0222198632 | 0.03388792 | 0.978 |
|  | LFM | F100 | 0.009664525 | -0.0187873113 | 0.03811636 | 0.877 |
|  | FCM | F100 | 0.034534940 | 0.0060831031 | 0.06298678 | 0.009* |
|  | LFM | S26 | 0.003830499 | -0.0242233900 | 0.03188439 | 0.995 |
|  | FCM | S26 | 0.028700914 | 0.0006470245 | 0.05675480 | 0.042* |
|  | FCM | LFM | 0.024870414 | -0.0035814222 | 0.05332225 | 0.115 |

**Note:**

- ‘*’ represents statistically significant, while no esthetics represent a non-statistically significant result.
- Substrates are represented by - RCM: raw cow milk, F100: F100 infant formula, S26: S26 infant formula, LFM: ultra-high temperature low-fat milk, and FCM: ultra-high temperature full-cream milk.
