# Supplementary material for: Species sorting shapes the divergence of a traditional fermented dairy-derived bacterial community with repeatable functionality during propagation with alternative substrates
Source: World J Microbiol Biotechnol. 2026 Apr 28;42(5):243. doi: 10.1007/s11274-026-04830-3 (PMC13124831; doi:10.1007/s11274-026-04830-3)
Supplement: Supplementary file 9 — (DOCX 16.6 KB) [file 11274_2026_4830_MOESM9_ESM.docx]

**Table S8** Assessment of pH variation following the propagation of the mabisi microbial community across varied substrate types. The statistical analysis was performed using the Kruskal-Wallis test, followed by Dunn’s pairwise comparisons, with *p*-values adjusted for multiple testing using the Benjamin-Hochberg method

| **Substrate variation** | **Chi-squired** | **Degree of freedom** | ***p*-value** |  |
| --- | --- | --- | --- | --- |
| Test: Kruskal-Wallis rank sum | 220.02 | 4 | 1.854051e-46 |  |
| Test: pairwise comparison (Dunn test) | **Substrate group 1** | **Substrate group 2** | **Z-values** | **Adjusted *p*-value** |
|  | F100 | FCM | 1.330 | 0.115 |
|  | F100 | LFM | 1.722 | 0.061 |
|  | FCM | LFM | 0.393 | 0.347 |
|  | F100 | RCM | -1.023 | 0.170 |
|  | FCM | RCM | -2.352 | 0.016* |
|  | LFM | RCM | -2.745 | 0.006* |
|  | F100 | S26 | 11.978 | < 0.001* |
|  | FCM | S26 | 10.648 | < 0.001* |
|  | LFM | S26 | 10.256 | < 0.001* |
|  | RCM | S26 | 13.001 | < 0.001* |

**Note:**

- ‘*’ represents statistical significance, and no esthetics represent a non-statistically significant result.
- Substrate types include raw cow milk (RCM), F100 infant formula (F100), S26 infant formula (S26), ultra-high temperature low-fat milk (LFM), and ultra-high temperature full-cream milk (FCM).
