# Supplementary material for: Species sorting shapes the divergence of a traditional fermented dairy-derived bacterial community with repeatable functionality during propagation with alternative substrates
Source: World J Microbiol Biotechnol. 2026 Apr 28;42(5):243. doi: 10.1007/s11274-026-04830-3 (PMC13124831; doi:10.1007/s11274-026-04830-3)
Supplement: Supplementary file 10 — (DOCX 13.7 KB) [file 11274_2026_4830_MOESM10_ESM.docx]

**Table S9** Assessment of pH variation by propagation phase following repeated transfer of mabisi microbial communities in varied substrates over time. The statistical analysis was conducted using the Wilcoxon rank sum test

| **Propagation phase** | **W statistic** | ***p*-value** |
| --- | --- | --- |
| Test: Wilcoxon rank sum test | 15774 | 1.397e-05* |

**Note:** ‘*’ represents statistical significance, and no esthetics represent a non-statistically significant result.
