# Supplementary material for: Species sorting shapes the divergence of a traditional fermented dairy-derived bacterial community with repeatable functionality during propagation with alternative substrates
Source: World J Microbiol Biotechnol. 2026 Apr 28;42(5):243. doi: 10.1007/s11274-026-04830-3 (PMC13124831; doi:10.1007/s11274-026-04830-3)
Supplement: Supplementary file 11 — (DOCX 14.5 KB) [file 11274_2026_4830_MOESM11_ESM.docx]

**Table S10** Assessment of pH by farm site following the repeated propagation of mabisi microbial community in varied substrates over time. The statistical analysis was performed using the Kruskal-Wallis test, followed by Dunn’s pairwise comparison, with *p*-values adjusted for multiple testing using the Benjamin-Hochberg method

| **Farm site** | **Chi-squared** | **Degree of freedom** | ***P* - value** |  |
| --- | --- | --- | --- | --- |
| Kruskal-Wallis rank sum | 23.68994 | 2 | 7.174545e-06* |  |
| pairwise comparison (Dunn test) | **Farm group 1** | **Farm group 2** | **Z-values** | **Adjusted**  ***p*-value** |
|  | Farm 1 | Farm 2 | 4.22 | < 0.001* |
|  | Farm 1 | Farm 3 | 0.01 | 0.496 |
|  | Farm 2 | Farm 3 | -4.21 | < 0.001* |

**Note:** ‘*’ represents statistical significance, and no esthetics represent a non-statistically significant result.
