# Supplementary material for: Species sorting shapes the divergence of a traditional fermented dairy-derived bacterial community with repeatable functionality during propagation with alternative substrates
Source: World J Microbiol Biotechnol. 2026 Apr 28;42(5):243. doi: 10.1007/s11274-026-04830-3 (PMC13124831; doi:10.1007/s11274-026-04830-3)
Supplement: Supplementary file 12 — (DOCX 17.3 KB) [file 11274_2026_4830_MOESM12_ESM.docx]

**Table S11** Analysis of consistency variation in samples following the repeated propagation of mabisi microbial communities in varied substrates at different farm sites over time. The statistical analysis was performed using the Kruskal-Wallis test (or Wilcoxon rank sum test when appropriate), followed by Dunn’s pairwise comparison, with *p*-values adjusted for multiple testing using the Benjamin-Hochberg method

| **Substrate variation** | **Chi-Square** | **Degrees of freedom** | ***p*-value** |  |
| --- | --- | --- | --- | --- |
| Test: Kruskal-Wallis rank sum | 137.51 | 4 | 9.64372e-29 |  |
| Test: Pairwise comparison (Dunn’s test) | **Substrate group 1** | **Substrate group 2** | **Z-value** | **Adjusted**  ***p*-value** |
|  | F100 | FCM | 5.645 | < 0.001* |
|  | F100 | LFM | 0.278 | 0.3905 |
|  | FCM | LFM | -5.367 | < 0.001* |
|  | F100 | RCM | -3.799 | < 0.001* |
|  | FCM | RCM | -9.444 | < 0.001* |
|  | LFM | RCM | -4.077 | < 0.001* |
|  | F100 | S26 | -4.890 | < 0.001* |
|  | FCM | S26 | -10.535 | < 0.001* |
|  | LFM | S26 | -5.168 | < 0.001* |
|  | RCM | S26 | -1.091 | 0.1529 |
| **Propagation phase** | **W statistic** |  | ***p*-value** |  |
| Test: Wilcoxon rank sum test | 20748 |  | 0.6685 |  |
| **Farm site** | **Chi squire test** | **Degree of freedom** | ***p*-value** |  |
| Test: Kruskal Wallis rank sum | 0.65527 | 2 | 0.7206 |  |

**Note:**

- ‘*’ represents statistical significance, and no esthetics represent a non-statistically significant result.
- Substrate types include raw cow milk (RCM), F100 infant formula (F100), S26 infant formula (S26), ultra-high temperature low-fat milk (LFM), and ultra-high temperature full-cream milk (FCM).
